# Transcriptome Analysis Reveals Novel Insights into the Hyperaccumulator *Phytolacca acinosa* Roxb. Responses to Cadmium Stress

**DOI:** 10.3390/plants13020297

**Published:** 2024-01-18

**Authors:** Qin Xie, Wentao Deng, Yi Su, Liying Ma, Haijun Yang, Feihong Yao, Wanhuang Lin

**Affiliations:** 1 College of Pharmacy, Xiangnan University, Chenzhou 423099, China; xieqin@stu.hunau.edu.cn (Q.X.);; 2 Hunan Provincial Key Laboratory of Phytohormones and Growth Development, Hunan Agricultural University, Changsha 410128, China; 3 College of Resources and Environment, Hunan Agricultural University, Changsha 410128, China

**Keywords:** *Phytolacca acinosa* Roxb., cadmium stress, phytoremediation, transcriptome, regulatory mechanism

## Abstract

Cadmium (Cd) is a highly toxic heavy metal that causes serious damage to plant and human health. *Phytolacca acinosa* Roxb. has a large amount of aboveground biomass and a rapid growth rate, and it has been identified as a novel type of Cd hyperaccumulator that can be harnessed for phytoremediation. However, the molecular mechanisms underlying the response of *P. acinosa* to Cd^2+^ stress remain largely unclear. In this study, the phenotype, biochemical, and physiological traits of *P. acinosa* seeds and seedlings were analyzed under different concentrations of Cd^2+^ treatments. The results showed higher Cd^2+^ tolerance of *P. acinosa* compared to common plants. Meanwhile, the Cd^2+^ content in shoots reached 449 mg/kg under 10 mg/L Cd^2+^ treatment, which was obviously higher than the threshold for Cd hyperaccumulators. To investigate the molecular mechanism underlying the adaptability of *P. acinosa* to Cd stress, RNA-Seq was used to examine transcriptional responses of *P. acinosa* to Cd stress. Transcriptome analysis found that 61 genes encoding TFs, 48 cell wall-related genes, 35 secondary metabolism-related genes, 133 membrane proteins and ion transporters, and 96 defense system-related genes were differentially expressed under Cd^2+^ stress, indicating that a series of genes were involved in Cd^2+^ stress, forming a complex signaling regulatory mechanism. These results provide new scientific evidence for elucidating the regulatory mechanisms of *P. acinosa* response to Cd^2+^ stress and new clues for the molecular breeding of heavy metal phytoremediation.

## 1. Introduction

Heavy metal pollution is a serious environmental problem that causes significant damage to plant and human health worldwide [1]. In recent years, the high mobility, toxicity, and non-degradable characteristics of heavy metals have raised concerns concerning their detrimental impact on the environment. It is now understood that heavy metal-polluted soils pose serious environmental issues and threaten agriculture and food safety [2]. Among the heavy metals, Cd is one of the most toxic to living organisms; it is not degradable and can easily accumulate in soil [3,4]. In China, the heavy metal pollution rate of farmland soil has reached 19.4% and is mainly caused by Cd [5]. Excessive accumulation of Cd has many deleterious effects on plants, such as reducing biomass, reducing carotenoid and chlorophyll content, reducing leaf surface, reducing photosynthesis rates, and increasing protease activity, resulting in plant growth retardation and even death [6,7,8]. Moreover, due to being highly mobile and soluble, Cd can accumulate in the human body through the intake of grains and vegetables planted in Cd-polluted soil and cause serious harm to human health [9,10]. Therefore, there is an urgent need to find effective solutions that can mitigate the risks posed by Cd-polluted soils. While physical and chemical methods have been developed for soil remediation, their application is expensive and can sometimes introduce new pollutants into the soil [11].

Phytoremediation is an efficient and effective biotechnology to extract heavy metals from soil via harvestable portions of plants [12,13]. This approach has emerged as a natural and environmentally friendly method, and it has attracted significant attention in recent years for its low cost of implementation and environmental benefits as it is more likely to be acceptable to the public than other traditional methods [14]. To successfully remediate heavy metal-polluted soil using phytoremediation, the selected plants must possess a significant amount of aboveground biomass, a rapid growth rate, and a high capacity to accumulate and tolerate contaminants in their harvestable parts [11]. However, many naturally occurring hyperaccumulators either exhibit slow growth, resulting in low aboveground plant biomass, or are poorly adapted to a variety of environmental conditions, limiting their application in phytoremediation efforts [15]. It is now understood that plants that accumulate more than 100 mg/kg Cd in leaves or shoots can be used as candidate plants for Cd phytoremediation [16]. While some Cd hyperaccumulators have been documented, such as *Noccaea caerulescens* [17], *Sedum alfredii* [18], *Viola baoshanensis* [19], and *Solanum nigrum* [20], their small biomass and slow growth rate have limited their application in Cd phytoremediation. 

*Phytolacca acinosa* Roxb. is a perennial herbaceous plant from southern China. Current evidence suggests that the aboveground part of *P. acinosa* can enrich a lot of heavy metals, especially Mn and Cd [21]. A recent study revealed that, in Cd-polluted soil with Cd concentrations higher than 50 mg/kg, the Cd content in leaves and stems of *P. acinosa* both exceeded 100 mg/kg (dry weight), and the translocation factor was greater than 1, which was defined as Cd hyperaccumulators [21]. Compared with other hyperaccumulators, *P. acinosa* has a large amount of aboveground biomass and a rapid growth rate, especially with a high bioaccumulation factor [22]. The above studies overlap in their assertion that *P. acinosa* is not only a potent plant for Cd phytoremediation but can also be used to study molecular mechanisms of Cd accumulation and tolerance in plant cells. 

Cd-hyperaccumulators have evolved various strategies for Cd detoxification, including metal exclusion, binding Cd to the cell wall, restricting Cd accumulation in sensitive tissues/organelles, sequestration in vacuoles, chelation by organic compounds, and biochemical defenses [11,23]. Previous research has provided some physiological evidence, but few studies have focused on the molecular mechanisms of Cd accumulation and tolerance in *P. acinosa*. It has been reported that the metal hyperaccumulation, tolerance, and detoxification mechanisms are controlled and regulated by several genes [11,24]. For example, in *Arabidopsis*, AtHMA4 plays an important role in the accumulation and/or tolerance of Cd [25,26], and the MYB4-MAN3-Mannose-MNB1 signaling cascade regulates Cd stress tolerance through the glutathione-dependent phytochelatin synthesis pathway [27]. Besides, as SpHMA3 is located in the tonoplast and is specialized for Cd^2+^ transport, it promotes Cd detoxification in young leaves and stems by sequestrating Cd^2+^ into the vacuoles in *Sedum plumbizincicola* [28]. In *Malus micromalus*, the up-regulation of *FRO2*-like genes and *NRAMP3* contribute to high Cd transport and accumulation abilities [29]. These findings provide a comprehensive understanding of Cd hyperaccumulation at the molecular level. Therefore, cloning and functional analysis of genes responsive to Cd stress will shed light on the molecular mechanisms underlying Cd hyperaccumulation in *P*. *acinosa*.

To gain insights into the molecular mechanisms of *P. acinosa* response to Cd^2+^ stress, the Cd-accumulating ability of *P*. *acinosa*, the transcriptome analysis combined with physiological and biochemical traits were used to explore important genes and pathways response to Cd^2+^ stress in *P. acinose*. Importantly, our findings will provide the theoretical basis for soil phytoremediation.

## 2. Results

### 2.1. Response of P. acinosa Seeds to Cd^2+^ Treatment

**Figure 1:**
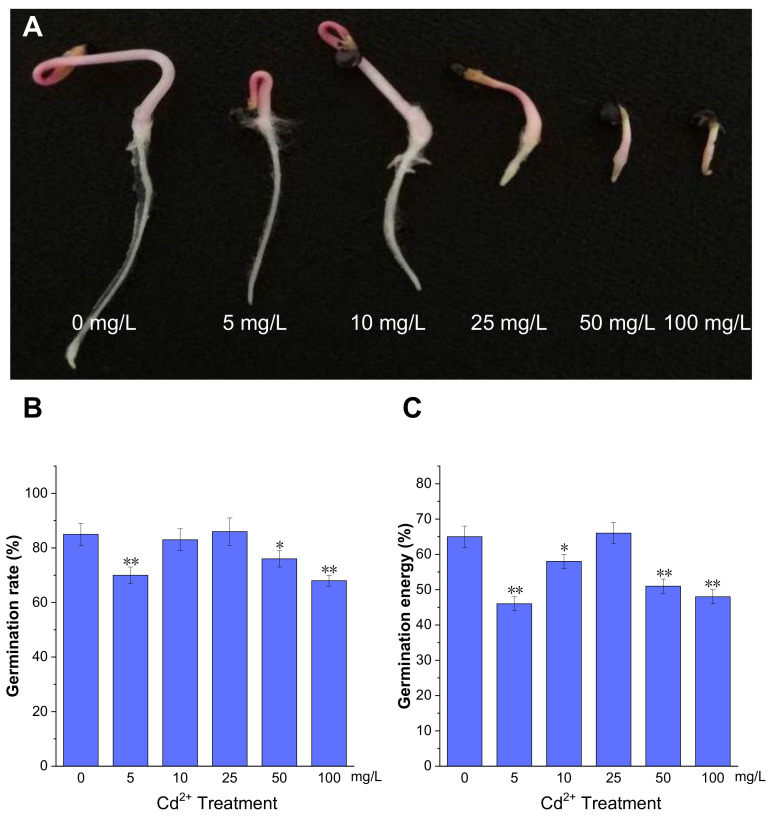
The germination of *P. acinosa* seeds under different Cd^2+^ treatments. (**A**) The phenotype of seed germination, (**B**) Germination rate, (**C**) Germination energy. Data shown as means ± SD of three biological replicates. Asterisks indicate a significant difference based on a Dunnett’s test. * significant difference at 5% level (*p* < 0.05); ** significant difference at 1% level (*p* < 0.01).

Seed germination is an important stage in the plant life cycle. To evaluate the effect of Cd^2+^ on *P. acinosa* seed germination, the seeds were treated with different Cd^2+^ concentrations during the germination period. The phenotype of *P. acinosa* seeds germination showed differences under 0, 5, 10, 25, 50, and 100 mg/L Cd^2+^ treatments. The results showed that, with increasing Cd^2+^ concentration, the root length gradually decreased compared to 0 mg/L Cd^2+^ treatment. Specifically, root lengths treated with 5 and 10 mg/L Cd^2+^ were slightly shorter than with 0 mg/L Cd^2+^ treatment, although the number of lateral roots and root hairs significantly increased. Moreover, under 25, 50, and 100 mg/L Cd^2+^ treatments, the growth of shoots and roots was significantly inhibited, and the seedlings died shortly after germination under 100 mg/L Cd^2+^ treatment (Figure 1A). 

Meanwhile, the germination energy was measured at 4 days post imbibition, and germination rate was measured at 7 days. The germination rates under 5, 50, and 100 mg/L Cd^2+^ treatments were 70%, 76%, and 68%, respectively, which were lower than with 0 mg/L Cd^2+^ treatment (85%). However, under 25 mg/L Cd^2+^ treatment, the germination rate was 86%, which was comparable to 0 mg/L Cd^2+^ treatment (Figure 1B). The germination energy of *P. acinosa* seeds under 5, 50, and 100 mg/L Cd^2+^ treatments were 46%, 51%, and 48%, respectively, which were lower than with 0 mg/L Cd^2+^ treatment (65%). However, the germination energy of *P. acinosa* seeds was 66% under 25 mg/L Cd^2+^ treatment, which was also comparable to 0 mg/L Cd^2+^ treatment (Figure 1C). Furthermore, under 10 mg/L Cd^2+^ treatment, the germination rate of *P. acinosa* seeds was 83%, which was not significantly different from 0 mg/L Cd^2+^ treatment (85%), and the germination energy was 58%, which was slightly lower than that under 0 mg/L Cd^2+^ treatment (65%), but higher than that under 5, 50, and 100 mg/L Cd^2+^ treatments.

The above results showed that the germination rate and germination energy of *P. acinosa* seeds were severely affected under 5, 50, and 100 mg/L Cd^2+^ treatments. However, under 25 mg/L Cd^2+^ treatment, the germination rate and germination energy were comparable to 0 mg/L Cd^2+^ treatment, but the growth of shoots and roots was significantly inhibited. Therefore, the combined analysis of *P. acinosa* seed phenotype with germination rate and energy substantiated that Cd^2+^ treatments of 5, 25, 50, and 100 mg/L could inhibit seed germination. However, under 10 mg/L Cd^2+^ treatment, mechanisms for defense and tolerance against Cd^2+^ stress were induced, leading to less impact on *P. acinosa* seed germination.

### 2.2. Response of P. acinosa Seedlings to Cd^2+^ Treatment

**Figure 2:**
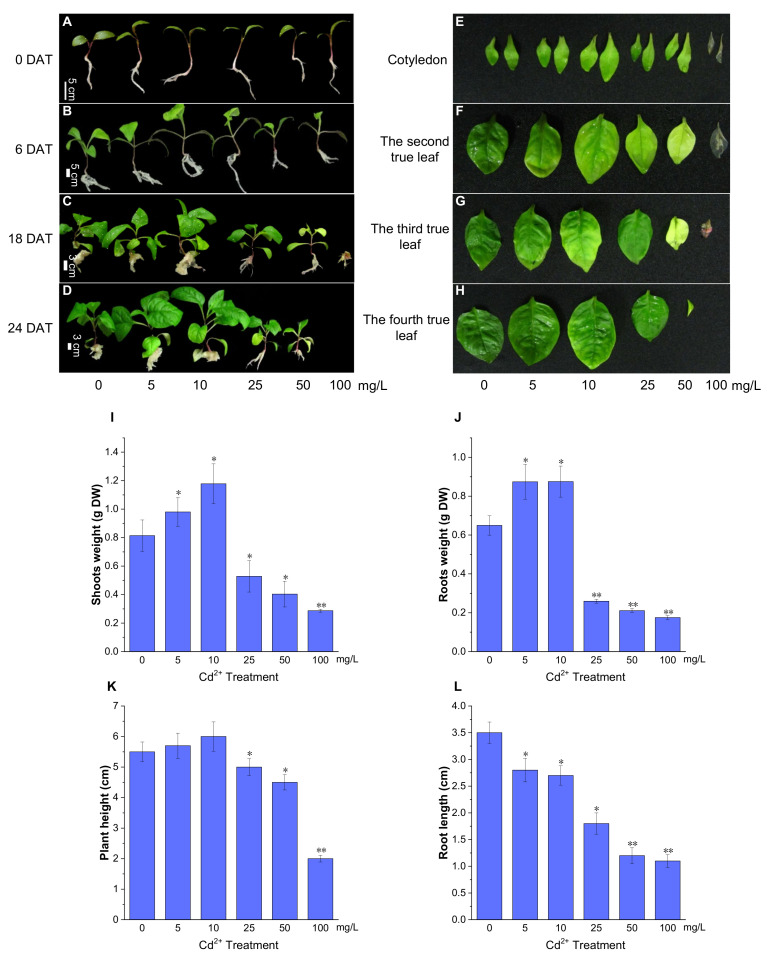
The growth status of *P. acinosa* seedlings under different Cd^2+^ treatments. (**A**–**D**) The phenotype of *P. acinosa* seedlings, (**E**–**H**) The phenotype of *P. acinosa* leaves, (**I**) Shoots weight, (**J**) Roots weight, (**K**) Plant height, (**L**) Root length. Data shown as means ± SD of three biological replicates (*n* = 30). Asterisks indicate a significant difference based on a Dunnett’s test. * significant difference at 5% level (*p* < 0.05); ** significant difference at 1% level (*p* < 0.01).

There was no phenotypic difference in *P. acinosa* seedlings under 0, 5, 10, 25, 50, and 100 mg/L Cd^2+^ treatments at 3 days after treatment (DAT). At 6 DAT, no significant differences were found in the seedlings under 0, 5, and 10 mg/L Cd^2+^ treatments. However, the seedlings under 50 and 100 mg/L Cd^2+^ treatments had smaller roots and shoots than those under 0 mg/L Cd^2+^ treatment, and the third true leaves grew slowly (Figure 2B). At 6 DAT, *P. acinosa* seedlings under 5 and 10 mg/L Cd^2+^ treatments exhibited better growth than those under 0 mg/L Cd^2+^ treatment. However, under 25, 50, and 100 mg/L Cd^2+^ treatments, the seedlings showed significant phenotypic damage with increased duration of Cd^2+^ treatment. At 18 DAT, *P. acinosa* seedlings under 25, 50, and 100 mg/L Cd^2+^ treatments exhibited more severely wilted leaves and a loss of strength. Moreover, *P. acinosa* seedlings under 100 mg/L Cd^2+^ treatment nearly died (Figure 2C). At 24 DAT, the seedlings under 5 and 10 mg/L Cd^2+^ treatments exhibited faster growth than those under 0 mg/L Cd^2+^ treatment. However, under 25 and 50 mg/L Cd^2+^ treatments, the seedlings were significantly worse than those under 0 mg/L Cd^2+^ treatment, and under 100 mg/L Cd^2+^ treatment, the seedlings completely died (Figure 2D). Observation of the leaves at 12 DAT showed that the leaf area was larger under 5 and 10 mg/L Cd^2+^ treatments than under 0 mg/L Cd^2+^ treatment. The leaves were small or even yellow under 25, 50, and 100 mg/L Cd^2+^ treatments, and the first pair of opposite leaves withered under 100 mg/L Cd^2+^ treatment (Figure 2E–H).

At 12 DAT, compared with 0 mg/L Cd^2+^ treatment, the dry biomass of the roots and shoots of *P. acinosa* seedlings (5 seedlings) was higher under 5 and 10 mg/L Cd^2+^ treatments but were significantly reduced under 25, 50, and 100 mg/L Cd^2+^ treatments (Figure 2I,J). The plant height of *P. acinosa* seedlings increased under 5 and 10 mg/L Cd^2+^ treatments but decreased under 25, 50, and 100 mg/L Cd^2+^ treatments. The root length of *P. acinosa* seedlings under 5, 10, 25, 50, and 100 mg/L Cd^2+^ treatments showed similar trends, which were shorter than that under 0 mg/L Cd^2+^ treatment (Figure 2K,L). Thus, the 5 and 10 mg/L Cd^2+^ treatments were considered to inhibit root elongation but promoted the development of the lateral root.

### 2.3. P. acinosa Has a High Cd^2+^ Accumulation Capacity

**Figure 3:**
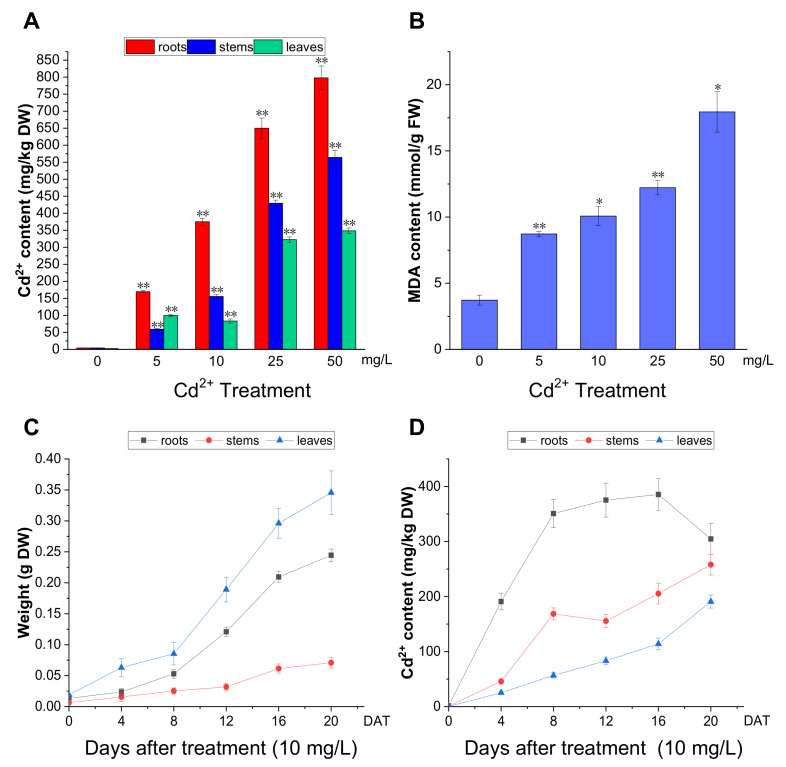
The Cd^2+^ accumulation in *P. acinosa* seedlings under different Cd^2+^ treatments. (**A**) Cd^2+^ content in roots, stems, and leaves of *P. acinosa* seedlings, (**B**) MDA content in roots of *P. acinosa* seedlings, (**C**) The weight of roots, stems, and leaves of *P. acinosa* seedlings at 0, 4, 8, 12, 16, and 20 DAT under 10 mg/L Cd^2+^ treatment, (**D**) Cd^2+^ content in roots, stems and leaves of *P. acinosa* seedlings at 0, 4, 8, 12, 16, and 20 DAT under 10 mg/L Cd^2+^ treatment. Data shown as means ± SD of three biological replicates (n = 30). Asterisks indicate a significant difference based on a Dunnett’s test. * significant difference at 5% level (*p* < 0.05); ** significant difference at 1% level (*p* < 0.01).

To further analyze the effect of Cd^2+^ on *P. acinosa*, we examined Cd^2+^ accumulation in *P. acinosa* seedlings. At 12 DAT, with increasing Cd^2+^ concentration, the Cd^2+^ content in the roots, stems, and leaves gradually increased. Except for the 5 mg/L treatment, the overall trend of Cd^2+^ content was roots > stems > leaves (Figure 3A). The results showed that the Cd^2+^ content in the roots, stems, and leaves of *P. acinosa* seedlings under 5, 10, 25, 50, and 100 mg/L Cd^2+^ treatments was significantly higher than that under 0 mg/L Cd^2+^ treatment, and *P. acinosa* seedlings could grow well under 5 and 10 mg/L Cd^2+^ treatments. Malondialdehyde (MDA) is a final product of lipid peroxidation, which is an important physiological index for measuring oxidative damage under stress [30]. Unlike under 0 mg/L Cd^2+^ treatment, the MDA content in the roots increased with increasing Cd^2+^ concentration (Figure 3B). The dry weight and Cd^2+^ content in *P. acinosa* seedlings under 10 mg/L treatment at 0, 4, 8, 12, 16, and 20 DAT were measured. As the duration of the 10 mg/L treatment was prolonged, the dry weight of roots, stems, and leaves gradually increased (Figure 3C), and the overall trend of Cd^2+^ content was roots > stems > leaves (Figure 3D). The Cd^2+^ content in roots reached its peak (385.43 mg/kg) at 16 DAT and then decreased, and at 20 DAT, the Cd^2+^ content in shoots reached 449 mg/kg, including 257.98 mg/kg and 190.90 mg/kg in stems and leaves, respectively (Figure 3D), which was significantly higher than the threshold for Cd hyperaccumulators (100 mg/kg Cd in leaves or shoots). In addition, the Cd^2+^ content in roots was 304.74 mg/kg at 20 DAT and the translocation factor was 1.48 (translocation factor > 1). This indicated that *P. acinosa* seedlings have a high Cd^2+^ tolerance and a strong ability to absorb and transport Cd^2+^ from the external environment.

### 2.4. De Novo Transcriptome Sequencing and Assembly

Total RNA was extracted from the seedling roots treated with 0 mg/L (CK) and 50 mg/L (Cd-T) Cd^2+^ for 48 h. De novo sequencing was performed on the Illumina HiSeq 4000 platform, and CK and Cd-T samples were conducted in three biological replicates. For the CK samples, 34,004,184, 42,566,702, and 42,821,964 clean reads with more than 95% Q30 bases were obtained (Table 1). For the Cd-T samples, 34,262,482, 38,182,134, and 46,290,484 clean reads with more than 95% Q30 bases were obtained (Table 1). The transcriptome sequencing data underwent quality analysis and were used for subsequent analysis.

The filtered clean reads from six samples were assembled using the Trinity program. The initial assembly obtained 255,468 transcripts with an N50 of 1781 bp. For these transcripts, the largest transcript was 17,759 bp, and the average length was 1171.11 bp. In total, 124,408 unigenes (N50 value = 1282) were obtained; the largest unigene was 17,759 bp, the smallest unigene was 301 bp, and the average length of unigenes was 914.36 bp (Table 2). The length of most of these unigenes (112,090, 90.99%) was ≤2000 bp. Overall, with increasing unigene length, the number of unigenes decreased, and unigene lengths between 301 and 400 bp accounted for the largest number (33,059, 26.57%) (Table 2).

### 2.5. Functional Annotation of Unigenes

To analyze the putative function of *P. acinosa*, unigenes were annotated against the public protein databases, including NR, Swiss-Prot, KOG, GO, and KEGG. In total, 59,642 unigenes were annotated and matched to one or more of the above-mentioned databases. Among these unigenes, 58,500 unigenes were detected and showed significance in the NR database. In the Swiss-Prot, KOG, GO, and KEGG databases, 37,801, 33,873, 35,683, and 9651 unigenes were detected and showed significance, respectively.

**Figure 4:**
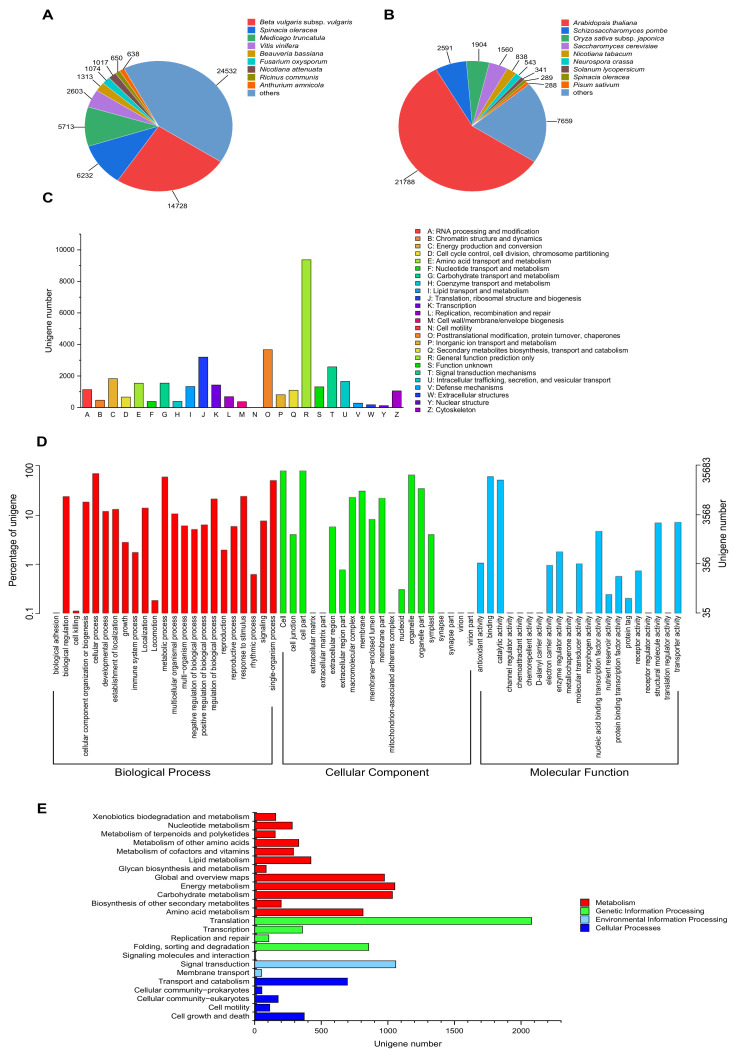
Functional annotation of unigenes. (**A**) NR database and (**B**) Swiss-Prot database, species distribution of top BLAST hits for each unigene with a cut-off E-value of 1.0 × 10^−5^. (**C**) KOG database, 33,873 unigenes were assigned to KOG classifications and divided into 25 specific categories. The y-axis represents the number of unigenes, and the x-axis represents 26 groups of KOG. (**D**) GO database, GO terms at level 2 were plotted, 35,683 unigenes were categorized into 65 functional groups. The y-axis represents the number and percentage of unigenes in the total number of genes annotation, and the x-axis represents the subcategories. (**E**) KEGG database, 9651 unigenes were assigned to “Metabolism”, “Genetic information processing”, “Environmental information processing”, and “Cellular processes”. The y-axis represents the subcategories, and the x-axis represents the number of unigenes.

Further analysis showed that in the NR database, the annotated sequences matched the sequences from *Beta vulgaris* subsp. vulgaris (14,728), *Spinacia oleracea* (6232), *Medicago truncatula* (5713), *Vitis vinifera* (2603), *Beauveria bassiana* (1313), *Fusarium oxysporum* (1074), *Nicotiana attenuata* (1017), *Ricinus communis* (650), *Anthurium amnicola* (638) and others (24,532) (Figure 4A). In the Swiss-Prot database, the annotated sequences shared homology with the sequences from *Arabidopsis thaliana* (21,788), *Schizosaccharomyces pombe* (2591), *Oryza sativa* subsp. *japonica* (1904), *Saccharomyces cerevisiae* (1560), *Nicotiana tabacum* (838), *Neurospora crassa* (543), *Solanum lycopersicum* (341), *Spinacia oleracea* (289), *Pisum sativum* (288) and others (7659) (Figure 4B).

In the KOG database, 33,873 unigenes were assigned to 25 KOG categories, with the largest category being “General function prediction only” (9370, 27.66%), followed by “Posttranslational modification, protein turnover, chaperones” (3664, 10.82%), and “Translation, ribosomal structure and biogenesis” (3197, 9.44%) (Figure 4C). In the GO database, 35,683 unigenes were categorized into 65 functional groups (level 2). For the “Biological process”, “Cellular component”, and “Molecular function” categories, the largest proportion of unigenes was clustered into “cell” (28,964, 81.17%), “cellular process” (25,502, 71.47%), and “binding” (22,151, 62.08%) (Figure 4D). In the KEGG database, 9651 unigenes were assigned to 4 groups, “Metabolism”, “Genetic information processing”, “Environmental information processing”, and “Cellular processes”. The unigenes were most significantly enriched in “Translation” (2079, 28.03%), followed by “Signal transduction” (1059, 14.28%), “Energy metabolism” (1052, 14.18%), and “Carbohydrate metabolism” (1034, 13.94%) (Figure 4E).

### 2.6. Differentially Expressed Genes (DEGs) Response to Cd^2+^ Stress in P. acinosa

**Figure 5:**
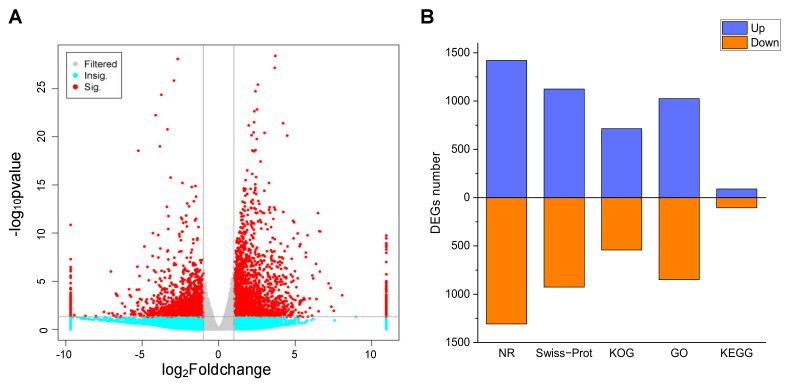
(**A**) Volcano analysis of DEGs between Cd-T and CK. Red dots represent significant; Cyan dots represent non-significant; Gray dots represents no significant change. (**B**) Annotation statistics of DEGs. Blue column represents up-regulated, red column represents down-regulated. DEGs were differentially expressed with statistical significance (|Log_2_FC(Cd-T/CK)| ≥ 1 and *p*-value ≤ 0.05).

Gene expression was estimated using the fragments per kilobase per million (FPKM) method. To identify the transcriptional changes during the *P. acinosa* response to Cd^2+^ stress, differential expression analysis between the CK and Cd-T samples was conducted, and 5061 DEGs were identified using DESeq (2012) software. The distribution of the DEGs was shown in Figure 5A. Among these DEGs, 2517 DEGs were up-regulated and 2544 DEGs were down-regulated. These DEGs were functionally annotated using the 5 databases. A total of 2743 DEGs (54.20%) were successfully annotated. The details were shown in Figure 5B.

### 2.7. GO Enrichment Analysis of DEGs

**Figure 6:**
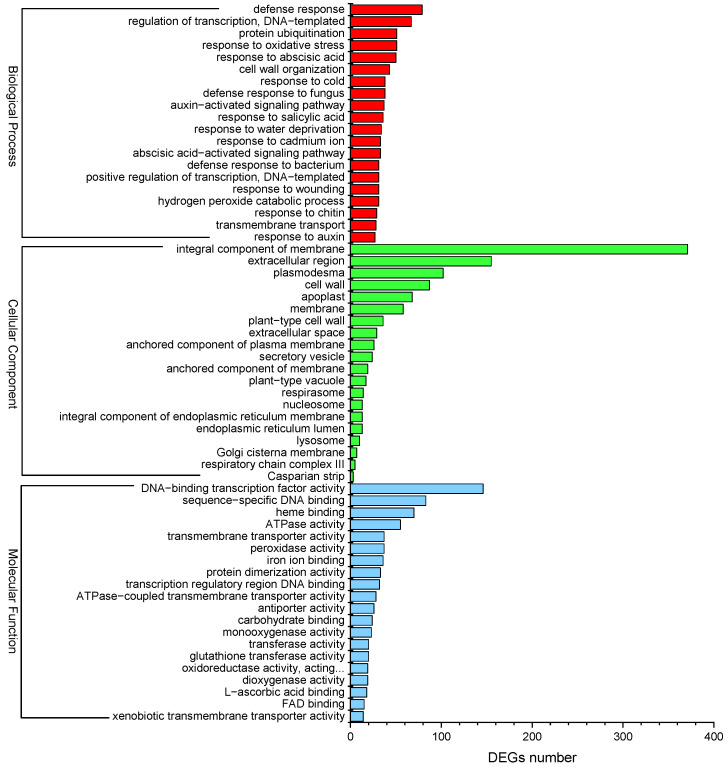
GO enrichment analysis of DEGs; the top 20 sub-categories are shown (*p*-value ≤ 0.05). Red column represents Biological process, green column represents Cellular component, blue column represents Molecular function. DEGs were differentially expressed with statistical significance (|Log_2_FC(Cd-T/CK)| ≥ 1 and *p*-value ≤ 0.05).

GO enrichment analysis was performed to evaluate the potential functions of DEGs responsive to Cd^2+^ stress. A total of 1874 DEGs were enriched in at least one term in the “Biological process”, “Cellular component”, and “Molecular function” categories. The top 20 sub-categories (*p*-value ≤ 0.05) were shown in Figure 6. In the “Biological process” category, “defense response” (GO:0006952) showed significant enrichment, followed by “regulation of transcription, DNA-templated” (GO:0006355) and “protein ubiquitination” (GO:0016567). In the “Cellular component”, the GO term “integral component of membrane” (GO:0016021) was significantly enriched, followed by “extracellular region” (GO:0005576) and “plasmodesma” (GO:0009506). Besides, the DEGs were significantly enriched in “Molecular function”, including “DNA-binding transcription factor activity” (GO:0003700), “sequence-specific DNA-binding” (GO:0043565), and “heme binding” (GO:0020037) (Figure 6). Moreover, “response to cadmium ion” (GO:0046686) and “peroxidase activity” (GO:0004601) were significantly enriched by several DEGs in the “Molecular function” category. “Response to oxidative stress” (GO:0006979) was enriched by some DEGs in the “Biological process”. “Abscisic acid-activated signaling pathway” (GO:0009738), “response to abscisic acid” (GO:0009737), “auxin-activated signaling pathway” (GO:0009734), and “response to salicylic acid” (GO:0009751) were also significantly enriched. The results of GO enrichment analysis may provide clues for understanding the transcriptomic profiles of genes involved in *P. acinosa* response to Cd^2+^ stress.

### 2.8. KEGG Pathway Enrichment Analysis of DEGs

**Figure 7:**
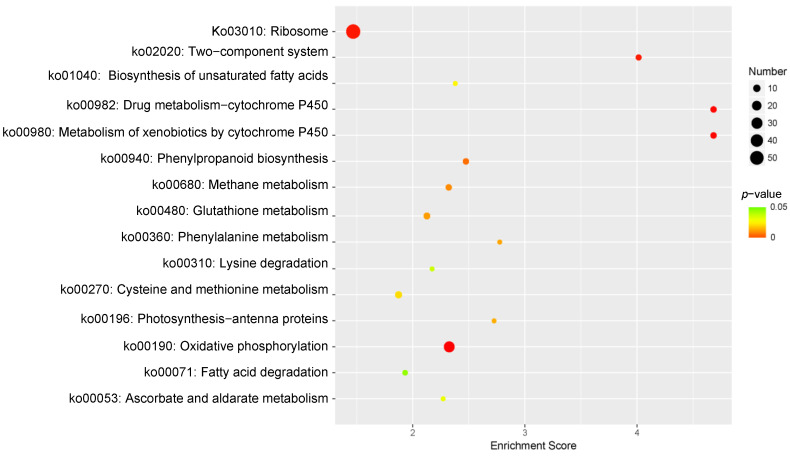
KEGG Pathway enrichment analysis of DEGs, 15 sub-categories are shown (*p*-value ≤ 0.05). DEGs were differentially expressed with statistical significance (|Log_2_FC(Cd-T/CK)| ≥ 1 and *p*-value ≤ 0.05).

KEGG pathway enrichment analysis was performed to identify the pathways responsive to Cd^2+^ stress in *P. acinosa.* In total, 15 KEGG pathways were significantly enriched (*p*-value ≤ 0.05) (Figure 7), among which “Ribosome” (ko03010) was associated with the highest number of DEGs (58 DEGs). These terms indicate the well-known basic alterations in protein turnover observed under Cd^2+^ stress conditions. Amino acid synthesis and the metabolism-related pathway “Cysteine and methionine metabolism” (ko00270), “Phenylalanine metabolism” (ko00360), and “Lysine degradation” (ko00310) were associated with nine, four, and four DEGs, respectively. “Oxidative phosphorylation” (ko00190), which is related to respiration, was associated with 28 DEGs. “Metabolism of xenobiotics by cytochrome P450” (ko00980) and “Drug metabolism-cytochrome P450” (ko00982) were related to substance metabolism, both of which enriched 7 DEGs. Fatty acid metabolism-related pathways “Fatty acid degradation” (ko00071) and “Biosynthesis of unsaturated fatty acids” (ko01040) were associated with five and four DEGs, respectively. Resistance-related pathway “Glutathione metabolism” (ko00480) was related to eight DEGs. These results suggested that, under Cd^2+^ stress, the change in the metabolism of plants involved in Cd^2+^ stress tolerance and the expression of genes was regulated by multiple signaling substances to adapt to the Cd^2+^ environment.

### 2.9. Transcription Factors Were Differentially Expressed under Cd^2+^ Stress

**Figure 8:**
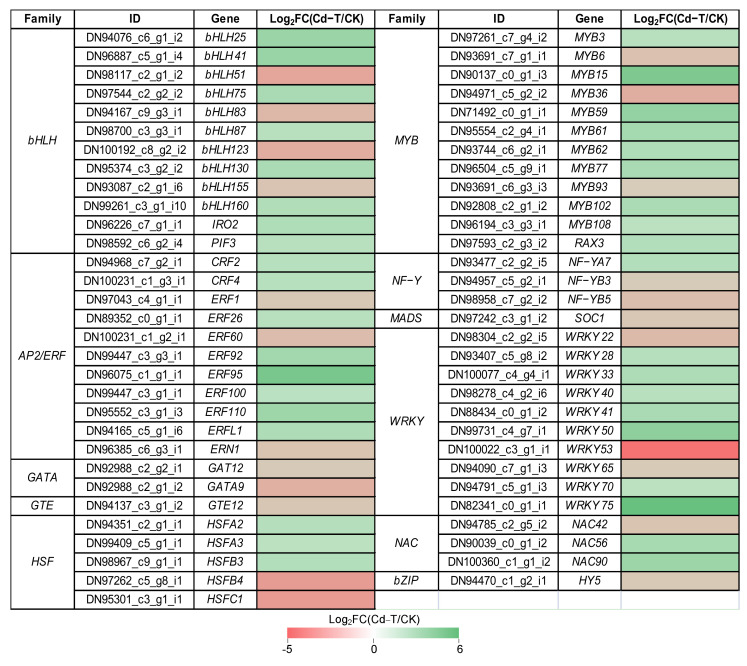
Expression profiles of DEGs encoding TFs were shown as log_2_FC(Cd-T/CK) levels. DEGs were differentially expressed with statistical significance (*p*-value ≤ 0.05 and |log_2_FC(Cd-T/CK)| ≥ 1).

Transcription factors (TFs) play an important role in regulating the expression of downstream genes. Under Cd^2+^ stress, 61 DEGs encoding TFs belonging to 11 families were found, including 38 DEGs that were up-regulated and 23 DEGs that were down-regulated, implying their functions in response to Cd^2+^ stress. The expression profiles of the DEGs encoding TFs were analyzed in detail (Figure 8). Overall, 12 *bHLH* (eight up-regulated and four down-regulated), 11 *AP2/ERF* (eight up-regulated and three down-regulated), two *GATA* (two down-regulated), one *GTE* (one down-regulated), five *HSF* (three up-regulated and two down-regulated), 12 *MYB* (nine up-regulated and three down-regulated), three *NF*-*Y* (one up-regulated and two down-regulated), one *MADS* (one down-regulated), 10 *WRKY* (seven up-regulated and three down-regulated), three *NAC* (two up-regulated and one down-regulated), and one *bZIP* (one down-regulated) were found. 

Many studies have shown that TFs are involved in external stress, such as *WRKY* family members, which are involved in the response to biotic and/or abiotic stresses. In this study, *WRKY75* showed the highest up-regulation (Log_2_FC(Cd-T/CK) = 5.7), and the homologs of *AtWRKY75* were identified as modulators of phosphate starvation responses [31]. *MYB* family members have been reported to be key factors in regulatory networks that regulate the development, metabolism, and responses to biotic and abiotic stresses [32]. *MYB15* was significantly up-regulated (Log_2_FC(Cd-T/CK) = 4.44), and the homologs of *AtMYB15* have been associated with the response to cold stress [33]. Besides, *MYB62* and *MYB108* showed increased expression under Cd^2+^ stress, and the homologs of *AtMYB62* have been associated with the response to phosphate starvation [34], while *AtMYB108* is reportedly involved in response to biotic and abiotic stress [35]. Previous studies have shown that NF-Y subunits could bind to bZIP to regulate the expression of ABRE-containing genes during adaptation to environmental stress [36]. In the present study, the expression of *NF*-*YA7* was increased under Cd^2+^ stress, and the homologs of *AtNF*-*YA7* have been reported to be sensitive to ABA and contribute to reducing the stress-induced damage by drought [37]. Previous studies have reported that bHLH family members are not only involved in plant growth and metabolism but also play an important role in plant response to stress, such as drought, salt, and cold stress [38]. *IRO2* (*bHLH* family member) was up-regulated under Cd^2+^ stress, and the homologs of *OsIRO2* were involved in Fe uptake under Fe-deficient conditions [39]. The results suggested that the expression patterns of TFs changed under Cd^2+^ stress, regulating the expression of downstream genes to adapt to the Cd^2+^ stress, and that the underlying regulation mechanism warrants further study.

### 2.10. Cell Wall-Related Genes Were Differentially Expressed under Cd^2+^ Stress

It is well-established that cell walls can effectively adsorb heavy metals to reduce their biological activities [40]. Sequestration of heavy metals in cell walls, vacuoles, and the Golgi apparatus is a detoxification process that reduces the direct contact of heavy metal ions with enzymes and metabolic compounds, thereby preventing the inactivation of enzymes and biochemical reactions [11]. Therefore, the cell wall plays an important role in heavy metal compartmentalization in hyperaccumulators [41]. To analyze the response of cell wall-related genes to Cd^2+^ stress, their expression patterns were examined. In this study, 48 DEGs were significantly associated with cell wall and were enriched in “cell wall biogenesis” (GO:0042546), “cell wall organization” (GO:0071555), “cell wall modification” (GO:0042545), “cellulose biosynthetic process” (GO:0030244), and “cellulose catabolic process” (GO:0030245). The expression profile is presented in Table 3. Regarding “cell wall biogenesis” and “cell wall organization”, most DEGs were down-regulated, suggesting that cell wall biosynthesis was significantly influenced by Cd^2+^ stress. 

Cell wall modification mediated by cell wall modifying enzymes helps plants adapt to external stress. Regarding “cell wall modification”, one pectinesterase gene (DN94343_c1_g2_i1) was up-regulated, and three pectinesterase inhibitor genes (DN92674_c4_g1_i2, DN92816_c2_g3_i2, and DN88314_c0_g1_i1) were down-regulated, contributing to the accumulation of pectonesterase. It has been reported that pectin can decompose into pectic acid and polygalacturonic acid through pectinesterase [42], both of which have a carboxyl group to facilitate Cd^2+^ binding to the cell wall [43]. The results showed that the differential expression of pectinesterase and pectinesterase inhibitor genes could promote Cd^2+^ binding to the cell wall. In addition, seven DEGs were up-regulated and two DEGs were down-regulated in “cellulose biosynthetic process”, and one DEG was up-regulated and four DEGs were down-regulated in “cellulose catabolic process”. The down-regulation of cellulose catabolic process-related genes and the up-regulation of cellulose biosynthetic process-related genes may contribute to the formation of the cell wall skeleton. 

### 2.11. Secondary Metabolism-Related Genes Were Differentially Expressed under Cd^2+^ Stress

Due to their chelating ability, the role of secondary metabolites in plant response to heavy metal stress has also received increasing attention [44]. In this study, according to the KEGG analysis, the expression of secondary metabolism-related genes was altered under Cd^2+^ stress. Specifically, 35 DEGs (24 up-regulated and 11 down-regulated) were associated with “flavonoid biosynthesis” (ko00941), “phenylpropanoid biosynthesis” (ko00940), “monoterpenoid biosynthesis” (ko00902), “cutin, suberine, and wax biosynthesis” (ko00073), “carotenoid biosynthesis” (ko00906), and “tropane, piperidine, and pyridine alkaloid biosynthesis” (ko00960) (Table 4). Next, “flavonoid biosynthesis” was associated with 16 DEGs, including 11 up-regulated DEGs and five down-regulated DEGs. Besides, “phenylpropanoid biosynthesis” was associated with eight DEGs, including five up-regulated DEGs and three down-regulated DEGs. In “cutin, suberine, and wax biosynthesis,” six DEGs were identified, including three up-regulated DEGs and three down-regulated DEGs. “Monoterpenoid biosynthesis”, “carotenoid biosynthesis”, and “Tropane, piperidine, and pyridine alkaloid biosynthesis” were associated with two, two, and one up-regulated DEGs, respectively. Among these DEGs, DN95456_c3_g5_i1 (flavonoid biosynthesis), DN86278_c0_g1_i1, and DN93922_c5_g2_i1 (phenylpropanoid biosynthesis) were significantly up-regulated, with fold changes of 6.37, 4.72, and 4.30 (Log_2_FC(Cd-T/CK) level), respectively. 

The differential expression of secondary metabolism-related genes under Cd^2+^ stress showed that secondary metabolism is one of the important mechanisms of *P. acinosa* response to Cd^2+^ stress, which could be related to the function of secondary metabolites in chelating Cd^2+^ to form Cd complexes and scavenging reactive oxygen species (ROS), significantly improving plant stress resistance.

### 2.12. Membrane Proteins and Ion Transporters Were Differentially Expressed under Cd^2+^ Stress

**Figure 9:**
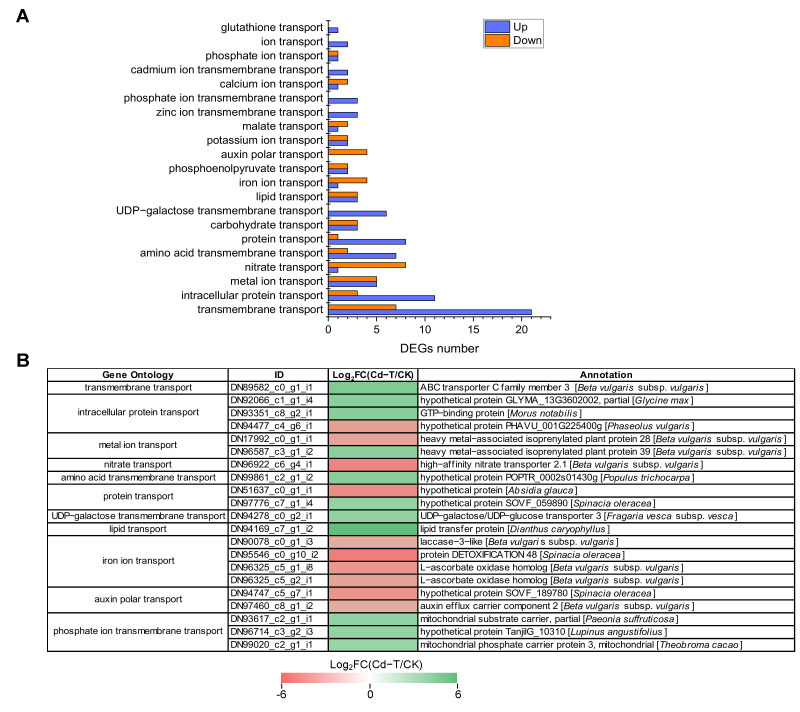
DEGs encoding membrane proteins and ion transporters under Cd^2+^ stress. (**A**) The number of DEGs encoding membrane proteins and ion transporters under Cd^2+^ stress. Blue column represents up-regulated DEGs, red column represents down-regulated DEGs. (**B**) Expression patterns of DEGs encoding membrane proteins and ion transporters under Cd^2+^ stress (top 20). DEGs were differentially expressed with statistical significance (|Log_2_FC(Cd-T/CK)| ≥ 1 and *p*-value ≤ 0.05).

Several genes encoding membrane proteins and ion transporters may exhibit changes when plants are exposed to Cd^2+^ stress. Based on the GO enrichment analysis, 133 DEGs encoding membrane proteins and ion transporters were enriched in 21 categories, including 84 up-regulated and 49 down-regulated DEGs (Table S1, Figure 9A). Further analysis found that 28 DEGs were enriched in “transmembrane transport” (GO:0055085), including 21 up-regulated DEGs and seven down-regulated DEGs. “Intracellular protein transport” (GO:0006886) was associated with 14 DEGs, including 11 up-regulated DEGs and three down-regulated DEGs. “Carbohydrate transport” (GO:0008643) and “metal ion transport” (GO:0030001) enriched six DEGs (three up-regulated and three down-regulated) and 10 DEGs (five up-regulated and five down-regulated), respectively. Furthermore, 17 other categories were associated with 75 DEGs (44 up-regulated and 31 down-regulated). Interestingly, two members of “cadmium ion transmembrane transport” were up-regulated and should be investigated in future studies. 

DEGs that were significantly up-regulated or down-regulated (top 20) are shown in Figure 9B. The expression of “lipid transport” (DN94169_c7_g1_i2) was up-regulated the most (Log_2_FC(Cd-T/CK) = 6.1), followed by “transmembrane transport” (DN89582_c0_g1_i1) and “amino acid transmembrane transport” (DN99861_c2_g1_i2). The “iron ion transport” (DN95546_c0_g10_i2) exhibited the most significant down-regulation (Log_2_FC(Cd-T/CK) = -5.04), followed by “nitrate transport” (DN96922_c6_g4_i1) and “protein transport” (DN51637_c0_g1_i1). These results suggested that many membrane proteins and ion transporters were involved in response to Cd^2+^ stress and contributed to the transport of macromolecules (protein and lipid) and small molecules (amino acid, ion, and harmful substance).

### 2.13. Defense System-Related Genes Were Differentially Expressed under Cd^2+^ Stress

**Figure 10:**
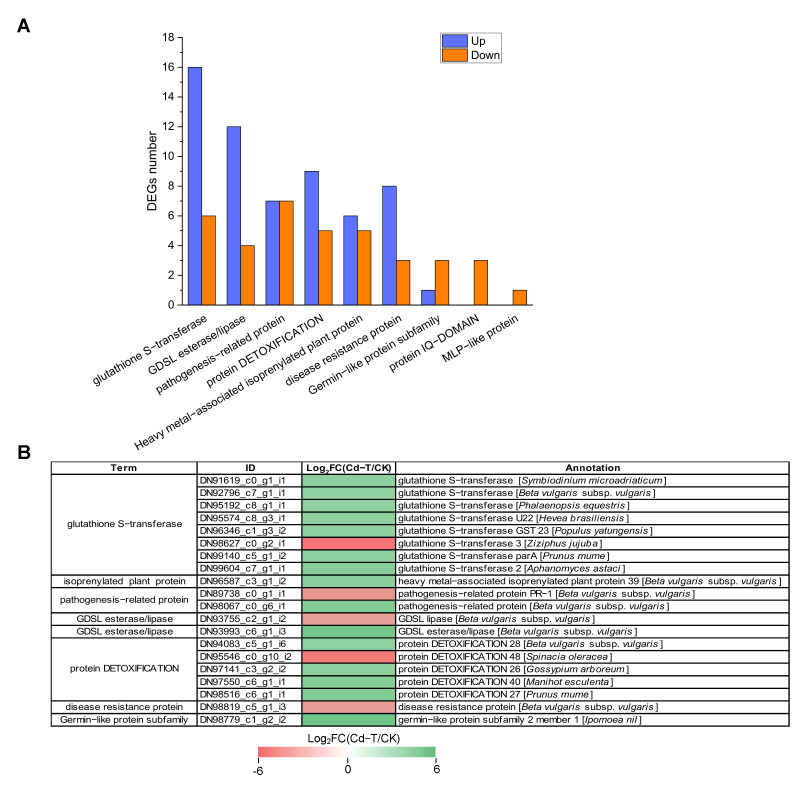
Expression profiles of defense system-related DEGs under Cd^2+^ stress. (**A**) The number of defense system-related DEGs under Cd^2+^ stress. Blue column represents up-regulated DEGs, red column represents down-regulated DEGs. (**B**) Expression patterns of defense system-related DEGs under Cd^2+^ stress (top 20). DEGs were differentially expressed with statistical significance (|Log_2_FC(Cd-T/CK)| ≥ 1 and *p*-value ≤ 0.05).

Protein denaturation and DNA damage can be induced by Cd^2+^ at the cellular level. Therefore, the production of defense proteins is necessary to sustain cellular homeostasis [45]. In this study, 96 defense-related DEGs were identified, with 59 up-regulated and 37 down-regulated DEGs (Table S2, Figure 10A). Among these DEGs, “glutathione S-transferase” was associated with the highest number of DEGs, including 16 up-regulated and six down-regulated DEGs. “GDSL esterase/lipase” contained 16 DEGs, with four up-regulated and 12 down-regulated DEGs. “Pathogenesis-related protein” was associated with 14 DEGs, with seven up-regulated and seven down-regulated DEGs. “Protein DETOXIFICATION” was also associated with 14 DEGs, including nine up-regulated and five down-regulated DEGs. Moreover, “heavy metal-associated isoprenylated plant protein”, “disease resistance protein”, “germin-like protein subfamily”, “protein IQ-DOMAIN”, and “MLP-like protein” were related to 11, 11, 4, 3, and 1 DEGs, respectively.

For a more comprehensive insight into the regulation of defense system-related genes, we presented the top 20 significantly up-regulated or down-regulated DEGs in Figure 10B. Among these DEGs, DN98779_c1_g2_i2 (germin-like protein subfamily), DN93993_c6_g1_i3 (GDSL esterase/lipase), and DN98067_c0_g6_i1 (pathogenesis-related protein) were significantly up-regulated with 5.34-, 4.94-, and 4.31-fold changes (Log_2_FC(Cd-T/CK) level), respectively. DN98627_c0_g2_i1 (glutathione S-transferase), DN95546_c0_g10_i2 (protein DETOXIFICATION), and DN89738_c0_g1_i1 (pathogenesis-related protein) were significantly down-regulated with 5.40-, 5.04-, and 3.80-fold changes (Log_2_FC(Cd-T/CK) level), respectively. Taken together, these results substantiated that several defense system-related DEGs were involved in response to Cd^2+^ stress, which helped alleviate the toxic effect of Cd^2+^ on *P. acinosa*. The defense mechanisms underlying the response of the significant defense system-related genes to Cd^2+^ stress warrant further study. 

### 2.14. Validation of the DEGs by Real-Time PCR Analysis

**Figure 11:**
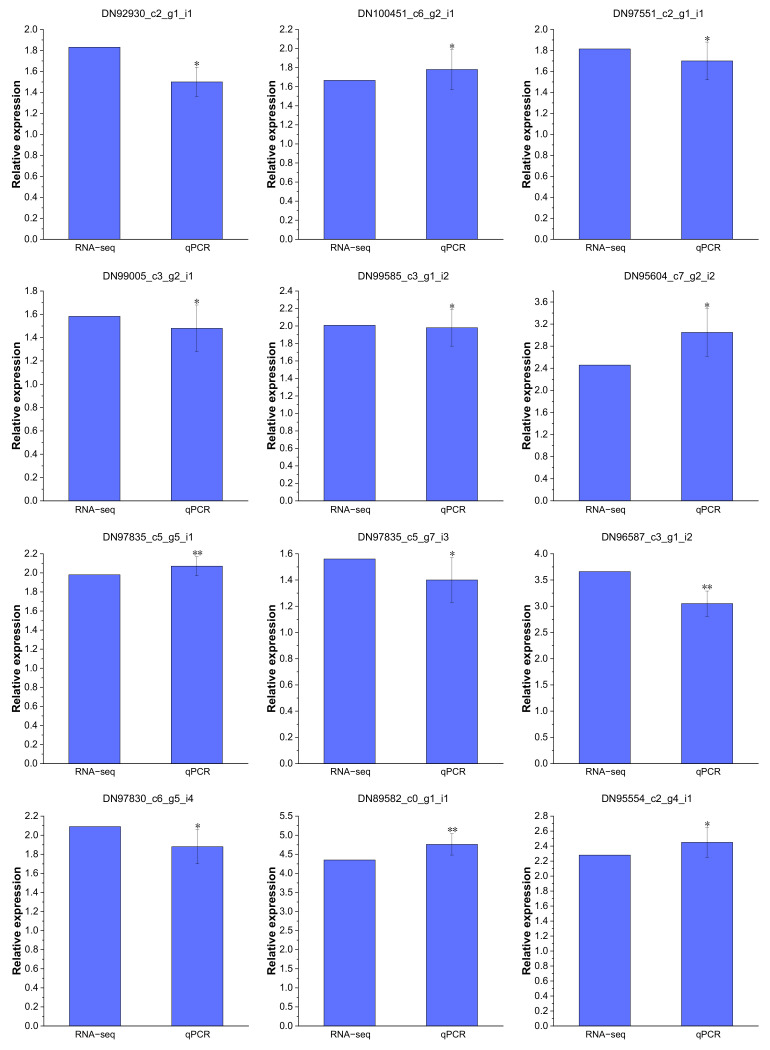
Expression patterns of 12 DEGs confirmed by real-time PCR, which are shown as log_2_Foldchange levels. DEGs were differentially expressed with statistical significance (|Log_2_FC(Cd-T/CK)| ≥ 1 and *p*-value ≤ 0.05). Data shown as means ± SD of three biological replicates. Asterisks indicate a significant difference based on a Dunnett’s test. * significant difference at 5% level (*p* < 0.05); ** significant difference at 1% level (*p* < 0.01).

Real-time PCR analysis was performed to validate the reliability of the RNA-Seq data. A total of nine Cd-regulated DEGs were selected to confirm the expression in *P. acinosa* roots under Cd^2+^ treatment, including six DEGs related to “transmembrane transport”, one DEG related to “zinc ion transmembrane transport”, two DEGs related to “cadmium ion transmembrane transport”, two DEGs related to “metal ion transport”, and one DEG related to “Transcription factors”. These results substantiated all selected DEGs were significantly up-regulated under Cd^2+^ treatment (Figure 11), and that the expression patterns were similar to the RNA-Seq data, indicating that the RNA-Seq data were reliable. 

## 3. Discussion

### 3.1. P. acinosa Can Be Used as a Candidate Plant for Cd Phytoremediation

Previous studies have shown that *Phytolacca americana* L. (*P. americana*) can accumulate large amounts of Cd in its aerial tissues. Under laboratory hydroponic conditions, the maximum Cd^2+^ content in aerial tissues of *P. americana* was 637 mg/kg, this remarkable ability to accumulate Cd appears to be a constitutive trait in this species, which makes it as a more suitable plants to remediate Cd polluted soil [22]. *P. acinosa,* found in various regions of southern China, has demonstrated remarkable adaptability to a wide spectrum of soil Cd concentrations and the ability to accumulate high Cd concentrations in its above-ground parts [21]. Plants that accumulate more than 100 mg/kg Cd in leaves or shoots can be used as candidate plants for Cd phytoremediation [16]. In this study, the Cd^2+^ content in shoots reached 449 mg/kg under 10 mg/L Cd^2+^ treatment at 20 DAT, which was significantly higher than the threshold for Cd hyperaccumulators, and the translocation factor was 1.48. Compared with other hyperaccumulators, *P. acinosa* exhibits a rapid growth rate and high above-ground biomass, indicating that *P. acinosa* has a high Cd^2+^ tolerance and a strong ability to absorb and transport Cd^2+^ from the external environment. Accordingly, it represents a candidate plant for Cd phytoremediation.

### 3.2. Numerous TFs Were Involved in P. acinosa Response to Cd^2+^ Stress

It is well-established that heavy metals can be toxic to plants through multiple damage mechanisms, such as cell membrane damage, photosynthetic rate reduction, ion homeostasis destruction, nutrient absorption inhibition, and nucleic acid and protein synthesis disorders, which eventually lead to plant growth stagnation and death [46]. The complex signaling regulatory mechanism in plants is important for heavy metal tolerance, and TFs play an important role in this regulatory network. Recent studies have identified a large number of TFs in plants that are involved in transcriptional regulation under Cd^2+^ stress [38,47]. 

The MYB family is one of the largest TF families in eukaryotes [48]. Previous studies demonstrated that *AtMYB4*, *AtMYB28*, *AtMYB43*, *AtMYB48*, *AtMYB72*, and *AtMYB124* were significantly induced in *Arabidopsis* under Cd^2+^ stress [49]. A recent study found that mutations in *OsMYB45* were associated with a Cd-sensitive phenotype in rice. Compared with wild-type leaves, the content of hydrogen peroxide in mutant leaves significantly increased, and catalase activity decreased [50,51]. The *bZIP* family has also been associated with resistance to various environmental stresses, including heavy metal stress. A study revealed that the *bZIP* member *BjCdR15* regulated Cd^2+^ uptake, transport, and accumulation in *Brassica juncea* [52]. In soybeans, *bZIP62* was significantly up-regulated under Cd^2+^ treatment, while *ThbZIP1* was down-regulated in *Tamarix hispida* [53,54]. Cd^2+^ also regulates the expression of the *AP2/ERF* family. The levels of *DREB1A* and *DREB1B* were up-regulated by Cd^2+^ stress in rice [55], while the expression of the *DRRB* family was inhibited by Cd^2+^ stress in *Solanum torvum* [56]. In this study, 61 DEGs encoding TFs belonging to 11 families were found under Cd^2+^ stress, including 12 *bHLH*, 11 *AP2/ERF*, 2 *GATA*, 1 *GTE*, 5 *HSF*, 12 *MYB*, 3 *NF*-*Y*, 1 *MADS*, 10 *WRKY*, 3 *NAC* and 1 *bZIP*. Among these TFs, the homologs of *AtWRKY75* [31], *AtMYB15* [33], *AtMYB62* [34], *AtMYB108* [35], *AtNF*-*YA7* [37], and *OsIRO2* [39] were involved in external stresses. The expression of *WRKY75*, *MYB15*, *MYB62*, *MYB108,* and *NF*-*YA7* were induced by Cd^2+^ stress in *P. acinosa*, which can be used as potential targets for promoting Cd^2+^ stress tolerance by modulating the expression of Cd-response genes in *P. acinosa*. More work is required to identify the significant TFs with drastic responses to conditions of Cd stress.

### 3.3. Multiple Mechanisms Were Involved in Cd^2+^ Stress Tolerance in P. acinosa

The cell wall is the first physical barrier restricting the entry of heavy metals into plant tissues, protecting plants from invasion by reducing the uptake of heavy metals or preventing them from entering the protoplasm [57]. The cell wall can serve as a “reservoir” for heavy metal accumulation by binding or coprecipitating heavy metals to improve stress tolerance [40]. In this study, 48 cell wall-related genes were differentially expressed under Cd^2+^ stress, these DEGs may contribute to the formation of the cell wall skeleton and promote Cd^2+^ binding to the cell wall.

The defense system is also activated to resist or reduce the toxic effects of stress under external stress. As one of the components of the antioxidant system, glutathione-S-transferase plays a major role in maintaining intracellular glutathione levels, detoxifying heavy metals, and improving plant tolerance [58]. The expression of most protein DETOXIFICATION genes, protein IQ-DOMAIN genes, disease resistance protein genes, and pathogenesis-related protein genes were identified to be up-regulated under Cd^2+^ stress in mung bean [59]. In this study, 35 secondary metabolism-related genes were differentially expressed under Cd^2+^ stress, these DEGs could be related to the function of secondary metabolites in chelating Cd^2+^ to form Cd complexes and scavenging ROS, significantly improving the Cd^2+^ stress tolerance of *P. acinosa*.

It is now understood that, when heavy metals are absorbed into plant cells, roots can quickly stimulate the stress response, alleviating heavy metal toxicity and improving plant tolerance through a series of complex defense and detoxification mechanisms. Secondary metabolic processes play an important role in response to Cd stress. Abiotic stresses can induce secondary metabolite formation and form a physical barrier, preventing osmotic stress and ROS damage [60]. Flavonoids have been identified as plant antioxidants involved in plant stress resistance, and Cd^2+^ can increase the gene expression of enzymes involved in flavonoid biosynthesis [61]. The synthesis of phenolic compounds can be used as a protective mechanism against heavy metal stress. Studies have shown that phenolic compounds have a strong chelation ability and are significantly increased in *Matricaria chamomilla* roots [62]. Under Cd^2+^ stress, the accumulation of isoflavones in *Medicago sativa* plays an important role in improving its long-term tolerance to Cd^2+^ stress [63]. In this study, 96 genes related to the defense system were differentially expressed under Cd^2+^ stress and may contribute to sustain cellular homeostasis.

### 3.4. ABC Transporters Were Involved in Regulating Cd^2+^ Stress

The function of the membrane transporter system is also important for plant responses to metal stress [64]. ATP-binding cassette transporters (ABC) are one of the largest protein families in plants, and they are responsible for transporting various metabolites, exogenous substances, and heavy metals, which play an important roles in heavy metal detoxification [65]. In this study, 21 ABC transporters were differentially expressed under Cd^2+^ stress, including 17 up-regulated and four down-regulated DEGs (Table S1). They were mainly distributed in the subfamilies of ABCB, ABCC, and ABCG.

Previous studies have reported that ABC transporters are involved in Cd stress tolerance in plants [66,67]. Moreover, ABC transporters were reported to play a role in Cd tolerance by Cd extrusion or vacuolar sequestration [67]. For example, in *Arabidopsis*, AtABCC1 and AtABCC2 participated in the sequestration of Cd [66], AtABCC3 was involved in Cd tolerance by transporting phytochelatin-Cd complexes into the vacuole [67], and AtABCC25 was involved in the transport and detoxification of Cd [68]. AtABCG36/AtPDR8 was an efflux transporter for Cd^2+^ or Cd conjugates and was important for Cd resistance in *Arabidopsis* [69]. OsABCG36 was required for Cd tolerance as it exported Cd or Cd conjugates from the root cells in rice [70]. In this study, *ABCC1*, *ABCC2*, *ABCC3,* and *ABCG36* were induced by Cd^2+^ stress in *P. acinosa*. Accordingly, we speculate that they may play a vital role in improving Cd^2+^ transport and detoxification, although their function and mechanism warrant further study.

## 4. Materials and Methods

### 4.1. Materials and Cd^2+^ Treatment

The seeds of *P. acinosa* were placed on 1/2 MS medium solidified by 0.8% agar at 30 °C. After 4 days, the seeds were placed at 22 °C under 16 h light/8 h dark. The germination rate and germination energy were calculated. After 7 days, the seedlings were transplanted into the culture bottle with 0, 5, 10, 25, 50, and 100 mg/L Cd^2+^ treatments. The Cd^2+^ was supplied as CdCl_2_ into 1/2 MS medium (with 0.8% agar), and seedlings were grown in the same environment. Each treatment included three biological replicates. The following equations were used: Germination rate (%) = number of germinated seeds on the 7th day/total number of seeds × 100% [71], germination energy (%) = number of germinated seeds on the 4th day/total number of seeds × 100% [72].

### 4.2. Analysis of Seedling Traits

The seedlings under 0, 5, 10, 25, 50, and 100 mg/L Cd^2+^ treatments were harvested at 0, 6, 12, 18, and 24 DAT, respectively. After washing the seedlings with tap water, the phenotypes of the seedlings were analyzed and compared. The plant height, root length, and dry weight (shoots and roots) were measured at 12 DAT under 0, 5, 10, 25, 50, and 100 mg/L Cd^2+^ treatments. Moreover, under 10 mg/L Cd^2+^ treatment, the seedlings were harvested at 0, 4, 8, 12, 16, and 20 DAT to measure dry weight and Cd^2+^ content. 

Drying method: the shoots (stems and leaves) and roots were separated and washed thoroughly with distilled water. Next, shoots and roots were dried at 105 °C for 30 min, and then at 80 °C until the weights remained constant. The dry weight of the shoots and roots was measured.

### 4.3. Cd^2+^ Content Analysis by ICP–MS

The shoots (stems and leaves) and roots were dried at 105 °C for 30 min, and then at 80 °C until the weights remained constant. The dry tissues of the roots, stems, and leaves were ground into powder, and then Cd^2+^ content analysis was conducted by ICP–MS according to the method we previously proposed [73]. The Cd^2+^ content was obtained according to the standard Cd curve. Each experiment included three biological replicates. Cd^2+^ translocation factor = Cd^2+^ content in shoots/Cd^2+^ content in roots.

### 4.4. Measurement of MDA Content

The seedlings were harvested at 12 DAT, and roots were used to measure MDA according to the methods previously proposed by Chen et al. [74]. Each experiment included three biological replicates.

### 4.5. Cd^2+^ Treatment for Transcriptome Sequencing

The *P. acinosa* seedlings were cultivated with quartz sand as previously described [73]. The 30-day-old *P. acinosa* seedlings were irrigated with 200 mL modified Hoagland solution with 0 and 50 mg/L Cd^2+^ treatments, and Cd^2+^ was supplied as CdCl_2_. Then, 48 h later, the roots from five identical seedlings under 0 and 50 mg/L Cd^2+^ treatments were collected as one sample. Next, the samples were wrapped with aluminum foil, immediately frozen with liquid nitrogen, and then stored at −80 °C for transcriptome analysis. All samples included three biological replicates.

### 4.6. Library Preparation and Next Generation Sequencing

The seedling roots treated with 0 and 50 mg/L Cd^2+^ for 48 h were used to extract total RNA and prepare the library as previously described [75]. The Total RNA was extracted from samples using Plant RNA Purification Reagent (Invitrogen, Carlsbad, CA, USA), and 1.5 μg RNA per sample was used as input material for the RNA sample preparations. Raw reads were processed to obtain clean reads by removing low quality bases at the 3’ end and the adapter sequences. Sequencing libraries were generated using NEBNext^®^ UltraTMRNA Library Prep Kit for Illumina^®^ (NEB, Beverly, MA, USA) following manufacturer’s recommendations, and index codes were added to attribute sequences to each sample. Then, the library preparations were sequenced on an Illumina Hiseq 4000 (Illumina, San Diego, CA, USA) and 150 bp paired-end reads were generated. 

### 4.7. De Novo Transcriptome Assembly and Functional Annotation

Raw data were processed using NGS QC Toolkit [76], and the reads containing ploy-N and the low-quality reads were removed to obtain the clean reads. Transcriptome de novo assembly was carried out using the Trinity program [77]. The read counts of each gene were obtained by Htseq-count [78], and the FPKM value of each gene was calculated using Cufflinks [79]. The unigenes were annotated against the NR, Swiss-Prot, and KOG databases using the BLAST algorithm with an E-value threshold of 1.0 × 10^•5^ [80]. GO annotation was performed using the Blast2GO 6.0 software (BioBam Bioinformatics SL, Boston, MA, USA) [81]. KEGG pathway annotation was performed by the online KEGG Automatic Annotation Server (http://www.genome.jp/kegg/ (accessed on 20 July 2022) [82].

### 4.8. Differentially Expression Analysis

The gene expression was estimated using the FPKM method. DEGs were identified between 0 mg/L (CK) and 50 mg/L (Cd-T) Cd^2+^ treatments using the DESeq (with replicates) functions estimateSizeFactors and nbinomTest [83]. |Log_2_FC(Cd-T/CK)| ≥ 1 and *p*-value ≤ 0.05 determined as DEGs were selected and analyzed by GO function and KEGG pathway enrichment. 

### 4.9. Real-Time PCR Analysis

Total RNA was extracted from the seedling roots treated with 0 and 50 mg/L Cd^2+^ for 48 h for real-time PCR analysis as previously described [75]. Three parallel reactions and three biological replications were performed. The actin gene of *P. acinosa* was used as an internal standard. Gene primer sequences used were shown in Table 5.

## 5. Conclusions

**Figure 12:**
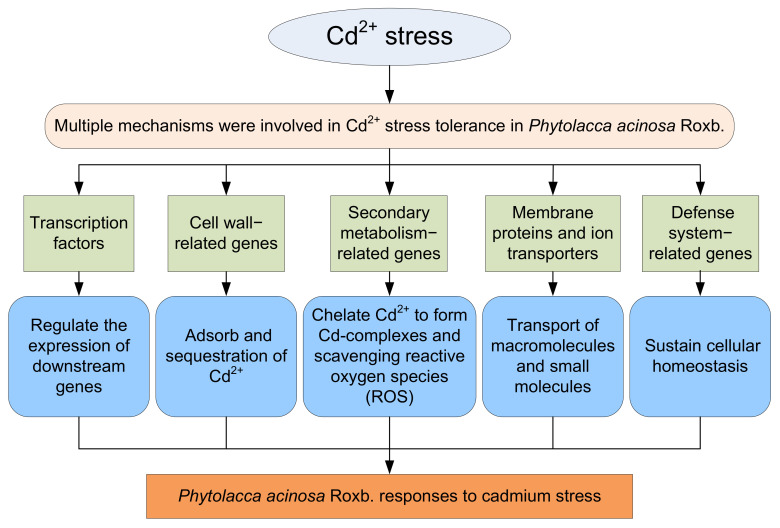
A proposed model of *Phytolacca acinosa* Roxb. response to Cd^2+^ stress.

In this study, the phenotype, biochemical, and physiological traits of *P. acinosa* seeds and seedlings under different Cd^2+^ treatments were analyzed. The results showed that the Cd^2+^ content in shoots of *P. acinosa* reached 449 mg/kg under 10 mg/L Cd^2+^ treatment, which was significantly higher than the threshold for Cd hyperaccumulators, indicating that *P. acinosa* has a high Cd^2+^ tolerance and a strong ability to absorb and transport Cd^2+^ from the external environment. Accordingly, it represents a candidate plant for Cd phytoremediation. To investigate the molecular mechanism underlying the adaptability of *P. acinosa* to Cd stress, RNA-Seq was used to examine transcriptional responses of *P. acinosa* to Cd stress. The filtered clean reads from six samples were assembled into 124,408 unigenes, and 59,642 unigenes were homologous with sequences in five public protein databases (NR, Swiss-Prot, KOG, GO, and KEGG). A total of 5061 DEGs (2517 up-regulated and 2544 down-regulated) were identified; then, 61 genes encoding TFs, 48 cell wall-related genes, 35 secondary metabolism-related genes, 133 membrane proteins and ion transporters, 96 defense system-related genes were differentially expressed under Cd^2+^ stress, indicating that a series of genes were involved in Cd^2+^ stress, forming a complex signaling regulatory mechanism. A model of *P. acinosa* response to Cd^2+^ stress was proposed in this study (Figure 12). Overall, our results provided new scientific evidence that elucidates the regulatory mechanisms of *P. acinosa* response to Cd^2+^ stress. In the future, these candidate DEGs should undergo functional studies to reveal the molecular mechanism of *P. acinosa* responses to Cd stress, providing new clues for the breeding of new cultivars with higher resistance as well as increased enrichment ability to Cd stress, which may further contribute to remediate Cd-polluted soil via phytoremediation technology.

## Figures and Tables

**Table 1:** Basic data of transcriptome sequencing.

Sample	Raw Reads Number	Raw Bases Number	Clean Reads Number	Clean Bases Number	Valid Bases (%)	Q20 (%)	Q30 (%)	GC (%)
CK1	34,397,898	5,159,684,700	34,004,184	5,075,043,936	98.35%	98.57%	95.56%	44.00%
CK2	42,980,760	6,447,114,000	42,566,702	6,353,506,726	98.54%	98.65%	95.74%	44.00%
CK3	43,298,432	6,494,764,800	42,821,964	6,391,566,154	98.41%	98.63%	95.67%	44.00%
Cd-T1	34,587,638	5,188,145,700	34,262,482	5,123,597,064	98.75%	98.60%	95.58%	44.00%
Cd-T2	38,393,362	4,759,004,300	38,182,134	4,682,860,231	98.40%	98.57%	95.55%	44.00%
Cd-T3	46,707,780	7,006,167,000	46,290,484	6,919,976,366	98.76%	98.65%	95.73%	44.00%

**Table 2:** Statistics of transcriptome assembly and predicted unigenes.

Type	Assembled Transcripts	Predicted Unigenes
Total sequence number	255,468	124,408
Total sequence base	299,181,020	113,753,609
Largest length (bp)	17,759	17,759
Smallest length (bp)	301	301
Average length (bp)	1171.11	914.36
N50 length (bp)	1781	1282

**Table 3:** Cell wall-related genes were differentially expressed under Cd^2+^ stress.

ID	Log_2_FC (Cd-T/CK)	Regulate	Annotation
**cell wall biogenesis**			
DN88120_c0_g1_i1	−2.01	Down	xyloglucan endotransglucosylase/hydrolase 2-like
DN92891_c3_g4_i1	1.66	Up	xyloglucan endotransglucosylase/hydrolase protein 23
DN92891_c3_g6_i1	−2.72	Down	xyloglucan endotransglucosylase/hydrolase protein22-like isoform X1
DN95917_c1_g1_i1	−2.41	Down	xyloglucan endotransglucosylase/hydrolase protein 23
DN96589_c1_g1_i2	2.41	Up	xyloglucan endotransglucosylase/hydrolase protein 6
DN96589_c1_g5_i1	−1.62	Down	xyloglucan endotransglucosylase/hydrolase protein 6
DN97081_c2_g1_i11	−1.21	Down	beta-arabinofuranosyltransferase RAY1 isoform X3
DN97099_c2_g1_i1	−1.47	Down	xyloglucan endotransglucosylase/hydrolase protein 7
DN98746_c2_g1_i4	−1.46	Down	xyloglucan endotransglucosylase/hydrolase 3
DN99640_c5_g1_i1	−1.05	Down	hypothetical protein SOVF_125280
DN88120_c0_g2_i1	−1.58	Down	hypothetical protein SOVF_073730
**cell wall organization**			
DN100083_c6_g2_i4	−1.11	Down	hypothetical protein SOVF_144400
DN108829_c0_g1_i1	1.62	Up	beta-1,3-galactosyltransferase 6
DN89571_c0_g1_i2	2.11	Up	rhamnogalacturonate lyase B
DN91042_c0_g1_i1	1.52	Up	polygalacturonase-like
DN91881_c0_g1_i1	−1.66	Down	hypothetical protein SOVF_175910
DN95973_c4_g1_i3	−1.26	Down	glucan endo-1,3-beta-glucosidase 13
DN95989_c1_g3_i3	1.28	Up	NAC transcription factor
DN96070_c1_g1_i10	1.97	Up	hypothetical protein BVRB_5g098360 isoform B
DN96441_c2_g2_i2	−1.32	Down	omega-hydroxypalmitate O-feruloyl transferase
DN96517_c3_g4_i1	−6.13	Down	Putative UDP-glucuronate:xylan alpha-glucuronosyltransferase 4
DN96603_c7_g3_i1	−2.65	Down	hypothetical protein BVRB_9g215820
DN96627_c2_g1_i1	−2.86	Down	hypothetical protein BVRB_9g215820
DN97041_c1_g1_i1	−1.09	Down	leucine-rich repeat extensin-like protein 4
DN98038_c4_g1_i5	−1.03	Down	beta-1,4-xylosyltransferase IRX9
DN91881_c0_g2_i1	−2.95	Down	casparian strip membrane protein 3
DN92988_c2_g2_i1	−1.20	Down	GATA transcription factor 12
DN92954_c10_g1_i1	−1.18	Down	uncharacterized protein LOC104819621
**cell wall modification**			
DN92674_c4_g1_i2	−1.39	Down	pectinesterase/pectinesterase inhibitor 21
DN92816_c2_g3_i2	−5.14	Down	pectinesterase/pectinesterase inhibitor 59
DN94343_c1_g1_i1	1.25	Up	KMT00424|hypothetical protein BVRB_9g217270
DN94343_c1_g2_i1	2.30	Up	putative pectinesterase 11
DN99628_c2_g1_i2	−1.35	Down	casparian strip membrane protein 2
DN88314_c0_g1_i1	−2.32	Down	pectinesterase/pectinesterase inhibitor 12
**cellulose biosynthetic process**			
DN94431_c0_g5_i2	1.95	Up	cellulose synthase-like protein G2
DN94789_c0_g4_i1	−5.27	Down	protein trichome birefringence-like 42
DN96659_c4_g1_i6	1.02	Up	hypothetical protein SOVF_137370
DN98575_c9_g1_i2	1.16	Up	hypothetical protein SOVF_143010
DN99723_c4_g4_i4	−1.43	Down	protein trichome birefringence-like 3
DN94431_c0_g6_i2	1.67	Up	hypothetical protein SOVF_076670
DN97763_c1_g5_i1	1.13	Up	hypothetical protein BVRB_5g111110
DN100016_c2_g3_i2	1.52	Up	hypothetical protein BVRB_6g142540
DN100016_c2_g2_i1	3.83	Up	hypothetical protein BVRB_6g142540
**cellulose catabolic process**			
DN94190_c4_g2_i1	−1.28	Down	endoglucanase 11
DN95890_c3_g1_i4	−1.23	Down	endoglucanase 6
DN96141_c3_g1_i4	1.19	Up	uncharacterized protein LOC104893418
DN95568_c3_g5_i1	−1.87	Down	endoglucanase 12
DN86253_c0_g1_i1	−2.54	Down	hypothetical protein SOVF_009160

DEGs were differentially expressed with statistical significance (|Log_2_FC(Cd-T/CK)| ≥ 1 and *p*-value ≤ 0.05).

**Table 4:** Secondary metabolism-related genes were differentially expressed under Cd^2+^ stress.

ID	Log_2_FC (Cd-T/CK)	Regulate	Annotation
**Flavonoid biosynthesis**			
DN110312_c0_g1_i1	−3.55	Down	chalcone synthase 2-like
DN91008_c0_g1_i2	−1.63	Down	protein SRG1-like
DN92655_c9_g1_i4	1.39	Up	hypothetical protein SOVF_156310
DN92655_c9_g6_i1	1.32	Up	hypothetical protein SOVF_156310
DN93138_c8_g4_i2	1.78	Up	hypothetical protein SOVF_168370
DN93301_c1_g1_i3	−2.93	Down	chalcone synthase 2-like
DN93562_c6_g3_i1	1.83	Up	chalcone synthase
DN95283_c6_g3_i1	1.53	Up	hypothetical protein SOVF_192090
DN95456_c3_g5_i1	6.37	Up	unnamed protein product
DN95974_c2_g2_i1	1.55	Up	hypothetical protein SOVF_200110
DN96124_c1_g1_i1	−1.15	Down	UDP-glycosyltransferase 79B6-like
DN96124_c1_g6_i2	1.60	Up	hypothetical protein SOVF_025340
DN96124_c1_g7_i2	1.28	Up	hypothetical protein SOVF_025340
DN96763_c2_g1_i1	2.09	Up	chalcone synthase
DN99228_c1_g1_i1	1.55	Up	flavanone-3-hydroxylase
DN99283_c4_g1_i1	−1.82	Down	hypothetical protein SOVF_168360
**phenylpropanoid biosynthesis**			
DN94161_c5_g1_i3	1.19	Up	cinnamyl alcohol dehydrogenase 1
DN98963_c5_g4_i1	−2.27	Down	hypothetical protein BVRB_8g195860 isoform B
DN74041_c0_g1_i1	−6.91	Down	class III peroxidase
DN86278_c0_g1_i1	4.72	Up	peroxidase 4
DN93922_c5_g2_i1	4.30	Up	hypothetical protein PRUPE_6G205600
DN96687_c1_g3_i2	1.06	Up	phenylalanine ammonia-lyase, partial
DN98188_c1_g4_i4	1.07	Up	phenylalanine ammonia-lyase
DN98963_c5_g4_i1	−2.27	Down	hypothetical protein BVRB_8g195860 isoform B
**Monoterpenoid biosynthesis**			
DN93319_c3_g1_i1	1.81	Up	(+)-neomenthol dehydrogenase-like, partial
DN96631_c4_g3_i1	2.21	Up	(+)-neomenthol dehydrogenase isoform X2
**Cutin, suberine and wax biosynthesis**			
DN96918_c3_g5_i1	2.40	Up	hypothetical protein SOVF_091970
DN93789_c3_g1_i1	−1.67	Down	long chain acyl-CoA synthetase 1
DN95362_c6_g2_i1	−2.06	Down	hypothetical protein BVRB_1g012280
DN99249_c1_g1_i1	2.44	Up	protein ECERIFERUM 3
DN99249_c1_g3_i4	3.35	Up	protein ECERIFERUM 3
DN98976_c3_g1_i1	−4.86	Down	omega-hydroxypalmitate O-feruloyl transferase
**carotenoid biosynthesis**			
DN98063_c4_g3_i1	1.04	Up	cyclo-DOPA 5-O-glucosyltransferase
DN20061_c0_g1_i1	2.16	Up	9-cis-epoxycarotenoid dioxygenase NCED5, chloroplastic
**Tropane, piperidine and pyridine alkaloid biosynthesis**			
DN98183_c5_g1_i1	1.22	Up	hypothetical protein SOVF_178490 isoform A

DEGs were differentially expressed with statistical significance (|Log_2_FC(Cd-T/CK)| ≥ 1 and *p*-value ≤ 0.05).

**Table 5:** The primer sequences for Real-time PCR.

ID	Primer Sequences 5′-3′
DN92930_c2_g1_i1	F: CCCTTTCACCCAGCCTAGTT
	R: TTGTACCAACGCCCCAAAGA
DN100451_c6_g2_i1	F: TGGCTTCATGACAATCCGCT
	R: AACGGAAGCCTAACCACTCG
DN97551_c2_g1_i1	F: GCGTGGACGGTATATGGGTT
	R: GCTACTGGCCCCATGAAGTT
DN99005_c3_g2_i1	F: TGCAACCGTAAGCGGATACT
	R: ATCCCCTCTAAACCCGTCCA
DN99585_c3_g1_i2	F: TTGCAGCCGCTTTCTATGGT
	R: GCCCTCTTCTGGTATGTGGG
DN95604_c7_g2_i2	F: TCATGTTGGCTTGTGCTGGA
	R: AGTTGAGCGCACAGGATTGA
DN97835_c5_g5_i1	F: AGAATGCGAAAGGCTTGGGA
	R: TGTGCTTGGTCCACACTCAG
DN97835_c5_g7_i3	F: GTAACCTGGGTCATCGGCTC
	R: TGGCTGCAATCGCATACCTT
DN96587_c3_g1_i2	F: AGTGTGGGGAGCCTAGTGAT
	R: GGTCTAAAGGACCCACACG
DN97830_c6_g5_i4	F: GTAACACCTCCATCTCCGCC
	R: AAAAGCCACCGGAAGAAGC
DN89582_c0_g1_i1	F: CCGAGACCCGTGTTTGTGTA
	R: CTGTTTTGGGCTTCACCAGC
DN95554_c2_g4_i1	F: TGTGGCCCACTCAACTAAGG
	R: AGATCCCCACCTGGATCAGA
Actin	F: TTGAGCAGGAATCGGAG
	R: TGCTGCTTCCATACCTATC

## Data Availability

The data presented in this study are available on request from the corresponding author.

## Supplementary Materials

The following supporting information can be downloaded at: https://www.mdpi.com/article/10.3390/plants13020297/s1, Table S1. Membrane proteins and ion transporters were differentially expressed under Cd^2+^ stress. Table S2. The defense system-related genes were differentially expressed under Cd^2+^ stress.
