# Supplementary material for: Transcriptome Analysis Reveals Novel Insights into the Hyperaccumulator Phytolacca acinosa Roxb. Responses to Cadmium Stress
Source: Plants (Basel). 2024 Jan 18;13(2):297. doi: 10.3390/plants13020297 (PMC10819451; doi:10.3390/plants13020297)
Supplement: Supplementary file 1 [file plants-13-00297-s001.zip › plants-2762271-supplementary.pdf]

Table S1. Membrane proteins and ion transporters were differentially expressed under Cd^{2+} stress.

ID	Log_2FC(Cd−T/CK)	Regulate	Annotation
**transmembrane transport**			
DN73613_c0_g1_i1	1.16	Up	ABC transporter B family member 15
DN99400_c3_g2_i6	1.01	Up	ABC transporter C family member 1
DN99625_c0_g4_i9	1.06	Up	ABC transporter C family member 10
DN99952_c3_g1_i1	1.45	Up	ABC transporter C family member 12
DN91343_c0_g1_i2	2.25	Up	protein PIN−LIKES 1−like
DN94691_c9_g1_i4	1.52	Up	ABC transporter C family member 15
DN93896_c5_g1_i6	-1.95	Down	SPX domain−containing membrane protein
DN99400_c3_g1_i1	1.24	Up	ABC transporter C family member 4
DN99952_c3_g4_i1	1.07	Up	ABC transporter C family member 7
DN94747_c5_g1_i1	-1.11	Down	probable auxin efflux carrier component 1c
DN94747_c5_g3_i1	-1.21	Down	Auxin efflux facilitator isoform 1
DN94949_c1_g1_i4	1.46	Up	protein PIN−LIKES 1 isoform X1
DN99952_c3_g2_i1	1.27	Up	ABC transporter C family member 2
DN89582_c0_g1_i1	4.35	Up	ABC transporter C family member 3
DN92930_c2_g1_i1	1.83	Up	ABC transporter C family member 4
DN100451_c6_g2_i1	1.67	Up	ABC transporter C family member 5
DN96857_c2_g3_i1	1.56	Up	ABC transporter C family member 6
DN94327_c10_g3_i2	-1.68	Down	ABC transporter C family member 8
DN99005_c2_g1_i1	1.19	Up	hypothetical protein SOVF_158430
DN96181_c0_g1_i4	1.09	Up	ABC transporter G family member 11
DN99339_c5_g2_i7	2.31	Up	pleiotropic drug resistance protein 1
DN95322_c3_g1_i9	-1.06	Down	ABC transporter G family member 14
DN97551_c2_g1_i1	1.81	Up	ABC transporter G family member 29
DN99005_c3_g2_i1	1.58	Up	ABC transporter G family member 35
DN99585_c3_g1_i2	2.01	Up	ABC transporter G family member 42
DN97551_c3_g5_i1	1.49	Up	ABC transporter G family member 36
DN100088_c5_g1_i1	-1.35	Down	ABC transporter G family member 44
DN96199_c1_g5_i1	-1.26	Down	ABC transporter G family member 8
**intracellular protein transport**			
DN23844_c0_g1_i1	-2.11	Down	hypothetical protein SPPG_00964
DN78745_c0_g1_i1	-1.92	Down	coatomer alpha subunit
DN92066_c1_g1_i4	3.80	Up	hypothetical protein GLYMA_13G3602002, partial
DN92658_c5_g5_i1	1.60	Up	ras−related protein RABA1f
DN93351_c8_g2_i1	3.77	Up	GTP−binding protein
DN93448_c1_g2_i4	1.07	Up	transmembrane emp24 domain−containing protein
DN94477_c4_g6_i1	-3.39	Down	hypothetical protein PHAVU_001G225400g
DN95501_c5_g1_i3	1.48	Up	hypothetical protein BVRB_7g169380
DN96568_c7_g3_i1	1.14	Up	clathrin light chain 2
DN96671_c0_g1_i8	1.19	Up	syntaxin−41−like
DN96884_c5_g2_i1	1.50	Up	transmembrane emp24 domain−containing protein
DN98442_c2_g6_i1	1.85	Up	transmembrane emp24 domain−containing protein
DN98862_c6_g2_i3	1.25	Up	rab9 effector protein with kelch motifs
DN99318_c5_g5_i1	1.67	Up	GTP−binding protein SAR1A
**carbohydrate transport**			
DN91678_c0_g2_i1	2.32	Up	bidirectional sugar transporter
DN93238_c8_g2_i2	-1.29	Down	putative glycerol−3−phosphate transporter 1
DN93238_c8_g4_i2	-2.17	Down	putative glycerol−3−phosphate transporter 1
DN95333_c6_g2_i4	1.03	Up	hexose carrier protein HEX6−like

DN95713_c1_g1_i1	1.61	Up	probable sugar phosphate/phosphate translocator
DN96328_c2_g1_i5	-2.49	Down	bidirectional sugar transporter SWEET7
**metal ion transport**			
DN17992_c0_g1_i1	-3.19	Down	heavy metal−associated isoprenylated plant protein 28
DN87382_c1_g1_i1	-2.60	Down	heavy metal−associated isoprenylated plant protein 26
DN93115_c2_g1_i4	1.92	Up	hypothetical protein SOVF_061260
DN96321_c5_g1_i2	-1.83	Down	hypothetical protein SOVF_105960
DN96587_c3_g1_i2	3.66	Up	heavy metal−associated isoprenylated plant protein 39
DN97075_c1_g3_i1	-1.37	Down	hypothetical protein SOVF_039310
DN97321_c4_g2_i1	1.31	Up	UPF0329 protein ECU05_1680/ECU11_0050 isoform X2
DN97830_c6_g5_i4	2.09	Up	heavy metal−associated isoprenylated plant protein 9
DN96943_c1_g1_i2	1.54	Up	heavy metal−associated isoprenylated plant protein 21
DN99220_c6_g4_i5	-1.54	Down	heavy metal−associated isoprenylated plant protein 21
**nitrate transport**			
DN95177_c5_g2_i3	-1.55	Down	protein NRT1/ PTR FAMILY 2.7−like
DN95557_c3_g1_i2	1.08	Up	protein NRT1/ PTR FAMILY 6.3
DN95557_c3_g7_i1	-1.77	Down	protein NRT1/ PTR FAMILY 6.3
DN95672_c4_g5_i2	-2.63	Down	high−affinity nitrate transporter 2.1
DN96549_c1_g4_i1	-1.58	Down	hypothetical protein SOVF_084270
DN96922_c6_g4_i1	-4.64	Down	high−affinity nitrate transporter 2.1
DN97597_c4_g2_i1	-1.74	Down	protein NRT1/ PTR FAMILY 1.2−like
DN98018_c4_g1_i1	-1.53	Down	hypothetical protein SOVF_002470, partial
DN98400_c0_g1_i2	-1.42	Down	high−affinity nitrate transporter 3.1−like
**amino acid transmembrane transport**			
DN86021_c0_g1_i1	-1.36	Down	vacuolar amino acid transporter 1
DN94304_c6_g2_i1	1.86	Up	sodium−coupled neutral amino acid transporter 6
DN94619_c5_g1_i3	1.57	Up	lysine histidine transporter−like 8
DN94619_c5_g3_i1	1.27	Up	lysine histidine transporter−like 8
DN95132_c2_g2_i11	1.12	Up	amino acid transporter ANTL1
DN96143_c6_g1_i3	1.47	Up	vacuolar amino acid transporter 1
DN97126_c0_g4_i2	-1.69	Down	hypothetical protein SOVF_046980
DN98331_c2_g1_i2	1.10	Up	probable amino acid permease 7
DN99861_c2_g1_i2	3.91	Up	hypothetical protein POPTR_0002s01430g
**protein transport**			
DN100306_c3_g3_i1	1.81	Up	oligopeptide transporter
DN51637_c0_g1_i1	-4.22	Down	hypothetical protein
DN71699_c0_g1_i1	1.06	Up	mitochondrial import receptor subunit TOM6 homolog
DN95390_c5_g6_i1	1.22	Up	AP−4 complex subunit sigma−like
DN95636_c2_g2_i2	1.62	Up	hypothetical protein M569_10648, partial
DN96451_c6_g3_i1	1.20	Up	Golgi SNAP receptor complex member 1−2
DN97776_c7_g1_i4	3.23	Up	hypothetical protein SOVF_059890
DN99260_c3_g4_i1	1.35	Up	protein NRT1/ PTR FAMILY 8.1
DN99450_c2_g1_i2	1.42	Up	protochlorophyllide−dependent translocon component 52, chloroplastic
**UDP−galactose transmembrane transport**			
DN94278_c0_g2_i1	3.17	Up	UDP−galactose/UDP−glucose transporter 3
DN97433_c2_g2_i1	1.59	Up	UDP−galactose/UDP−glucose transporter 3
DN97433_c2_g3_i2	1.53	Up	hypothetical protein SOVF_137860
DN97433_c2_g5_i2	1.42	Up	hypothetical protein SOVF_137860
DN99669_c6_g3_i1	1.55	Up	hypothetical protein SOVF_137860
DN99669_c6_g4_i1	1.64	Up	hypothetical protein SOVF_137860

**lipid transport**			
DN35578_c0_g1_i2	-1.15	Down	non−specific lipid−transfer protein 1
DN93937_c1_g1_i1	1.49	Up	uncharacterized protein LOC104899430 isoform X1
DN94169_c7_g1_i2	6.10	Up	lipid transfer protein
DN94286_c4_g1_i1	-1.11	Down	hypothetical protein BVRB_2g027210
DN94867_c7_g4_i5	1.18	Up	hypothetical protein SOVF_159400, partial
DN98490_c6_g1_i1	-1.62	Down	hypothetical protein BVRB_5g118370
**iron ion transport**			
DN90078_c0_g1_i3	-2.77	Down	laccase−3−like
DN95546_c0_g10_i2	-5.04	Down	hypothetical protein SOVF_107520
DN96325_c5_g1_i8	-3.84	Down	L−ascorbate oxidase homolog
DN96325_c5_g2_i1	-3.21	Down	L−ascorbate oxidase homolog
DN97693_c2_g2_i4	1.55	Up	hypothetical protein SOVF_044350
**phosphoenolpyruvate transport**			
DN100017_c9_g1_i1	-2.53	Down	Glucose−6−phosphate/phosphate translocator 2, chloroplastic
DN95207_c3_g2_i1	2.04	Up	hypothetical protein SOVF_039350
DN99610_c8_g1_i1	1.13	Up	phosphate translocator precursor (plastid)
DN99610_c8_g2_i2	-2.08	Down	plastidic glucose 6−phoaphate/phosphate translocator2
**auxin polar transport**			
DN94747_c5_g7_i1	-3.88	Down	hypothetical protein SOVF_189780
DN97460_c8_g1_i2	-2.77	Down	auxin efflux carrier component 2
DN97557_c4_g1_i3	-1.52	Down	thermospermine synthase ACAULIS5 isoform X2
DN98095_c3_g1_i1	-1.96	Down	thermospermine synthase ACAULIS5
**potassium ion transport**			
DN85103_c0_g1_i1	-2.32	Down	high affinity H+/K+ symporter
DN93830_c2_g1_i3	2.08	Up	cation/H(+) antiporter 18
DN95935_c1_g1_i1	-1.41	Down	potassium channel SKOR, partial
DN97635_c8_g2_i2	1.35	Up	putative potassium transporter protein, partial
**malate transport**			
DN94761_c6_g1_i4	1.19	Up	hypothetical protein SOVF_001540
DN96079_c6_g1_i3	-2.12	Down	hypothetical protein SOVF_132820
DN98313_c3_g1_i4	-1.70	Down	aluminum−activated malate transporter 10
**zinc ion transmembrane transport**			
DN93411_c8_g2_i1	1.39	Up	hypothetical protein SOVF_122270
DN95604_c7_g1_i2	2.62	Up	zinc transporter 2−like
DN95604_c7_g2_i2	2.46	Up	hypothetical protein SOVF_054580
**phosphate ion transmembrane transport**			
DN93617_c2_g1_i1	3.26	Up	mitochondrial substrate carrier, partial
DN96714_c3_g2_i3	3.14	Up	hypothetical protein TanjilG_10310
DN99020_c2_g1_i1	3.00	Up	mitochondrial phosphate carrier protein 3, mitochondrial
**calcium ion transport**			
DN93163_c5_g1_i2	-1.32	Down	glutamate receptor 3.2
DN93398_c3_g1_i2	1.08	Up	hypothetical protein BVRB_9g208360
DN95381_c3_g2_i2	-1.74	Down	glutamate receptor 2.6
**cadmium ion transmembrane transport**			
DN97835_c5_g5_i1	1.90	Up	metal transporter Nramp6 isoform X1
DN97835_c5_g7_i3	1.56	Up	hypothetical protein SOVF_145490
**phosphate ion transport**			
DN94768_c2_g2_i2	-2.35	Down	inorganic phosphate transporter 1−3
DN94768_c2_g7_i2	2.11	Up	inorganic phosphate transporter 1−4

**ion transport**			
DN93717_c6_g1_i1	1.43	Up	hypothetical protein SOVF_070720
DN95188_c2_g3_i1	1.11	Up	uncharacterized protein LOC104900151
**glutathione transport**			
DN96106_c1_g1_i5	1.95	Up	protein CLT1, chloroplastic

DEGs were differentially expressed with statistical significance ($|Log_2FC(Cd-T/CK)| \geq 1$ and P−value ≤ 0.05).

Table S2. The defense system-related genes were differentially expressed under Cd^{2+} stress.

ID	$Log_2FC(Cd-T/CK)$	Regulate	Annotation
**glutathione S−transferase**			
DN100569_c2_g3_i2	2.39	Up	glutathione S−transferase U19
DN100569_c2_g5_i1	-1.75	Down	glutathione S−transferase
DN58280_c0_g1_i1	-1.56	Down	glutathione S−transferase F13
DN91619_c0_g1_i1	3.52	Up	glutathione S−transferase
DN92796_c7_g1_i1	3.33	Up	glutathione S−transferase
DN92796_c7_g2_i1	2.35	Up	glutathione S−transferase
DN92923_c3_g1_i1	-1.80	Down	glutathione S−transferase U10
DN93049_c2_g1_i1	3.19	Up	glutathione S−transferase
DN93200_c3_g2_i1	1.34	Up	glutathione S−transferase U7
DN93253_c5_g1_i3	1.13	Up	glutathione S−transferase U8
DN93253_c5_g4_i3	-1.14	Down	glutathione S−transferase
DN95192_c8_g1_i1	3.36	Up	glutathione S−transferase
DN95574_c8_g3_i1	3.47	Up	glutathione S−transferase U22
DN95574_c8_g5_i1	1.56	Up	glutathione S−transferase
DN96346_c1_g3_i2	3.54	Up	glutathione S−transferase GST 23
DN98152_c0_g6_i2	1.66	Up	glutathione S−transferase
DN98453_c4_g1_i5	2.52	Up	glutathione S−transferase U7
DN98627_c0_g2_i1	-5.40	Down	glutathione S−transferase 3
DN99140_c5_g1_i2	3.44	Up	glutathione S−transferase parA
DN99522_c5_g2_i2	-1.64	Down	glutathione S−transferase U17
DN99604_c7_g1_i1	3.92	Up	glutathione S−transferase 2
DN99963_c4_g7_i1	1.69	Up	glutathione S−transferase DHAR2
**isoprenylated plant protein/metal ion transport**			
DN17992_c0_g1_i1	-3.19	Down	heavy metal−associated isoprenylated plant protein 28
DN85810_c0_g1_i2	-2.98	Down	heavy metal−associated isoprenylated plant protein 47
DN87382_c1_g1_i1	-2.60	Down	heavy metal−associated isoprenylated plant protein 26
DN93115_c2_g1_i4	1.92	Up	heavy metal−associated isoprenylated plant protein
DN96587_c3_g1_i2	3.66	Up	heavy metal−associated isoprenylated plant protein 39
DN97075_c1_g3_i1	-1.37	Down	heavy metal−associated isoprenylated plant protein 32
DN97321_c4_g2_i1	1.31	Up	heavy metal−associated isoprenylated plant protein
DN97825_c1_g3_i1	2.19	Up	heavy metal−associated isoprenylated plant protein 12
DN97830_c6_g5_i4	2.09	Up	heavy metal−associated isoprenylated plant protein 9
DN96943_c1_g1_i2	1.54	Up	heavy metal−associated isoprenylated plant protein
DN99220_c6_g4_i5	-1.54	Down	heavy metal−associated isoprenylated plant protein 21
**pathogenesis−related protein**			
DN87315_c0_g1_i1	-2.37	Down	pathogenesis−related protein PR−1
DN89659_c0_g1_i1	1.49	Up	pathogenesis−related protein 1B
DN89738_c0_g1_i1	-3.80	Down	pathogenesis−related protein PR−1

DN92106_c0_g2_i1	3.26	Up	pathogenesis−related protein bet V I family protein
DN94410_c1_g1_i2	-2.32	Down	pathogenesis−related protein
DN94410_c1_g2_i2	-1.52	Down	pathogenesis−related protein
DN94410_c1_g3_i1	-1.41	Down	pathogenesis−related protein STH−2
DN94410_c1_g6_i1	-1.98	Down	pathogenesis−related protein
DN94498_c3_g4_i3	1.18	Up	pathogenesis−related protein STH−21
DN94498_c3_g5_i1	2.88	Up	pathogenesis−related protein STH−21
DN96477_c4_g1_i2	-1.48	Down	pathogenesis−related protein
DN98067_c0_g3_i1	1.86	Up	pathogenesis−related protein PRB1−2
DN98067_c0_g5_i2	3.33	Up	pathogenesis−related protein 1B
DN98067_c0_g6_i1	4.31	Up	pathogenesis−related protein
**GDSL esterase/lipase**			
DN93398_c4_g1_i1	-1.22	Down	GDSL esterase/lipase 5
DN93755_c2_g1_i2	-3.39	Down	GDSL lipase
DN93993_c6_g1_i3	4.94	Up	GDSL esterase/lipase
DN94093_c6_g1_i2	-3.09	Down	GDSL esterase/lipase
DN94125_c2_g1_i3	1.65	Up	GDSL esterase/lipase
DN95501_c5_g1_i3	1.48	Up	GDSL esterase/lipase−like protein 25
DN95845_c4_g5_i7	1.27	Up	GDSL esterase/lipase
DN96158_c2_g1_i2	1.27	Up	GDSL esterase/lipase 1
DN96158_c2_g3_i1	1.90	Up	GDSL esterase/lipase 1
DN97209_c6_g1_i6	1.57	Up	GDSL esterase/lipase 7
DN97871_c1_g2_i2	1.02	Up	GDSL esterase/lipase 2
DN97871_c1_g7_i1	2.27	Up	GDSL lipase 2
DN99552_c7_g1_i2	1.99	Up	GDSL esterase/lipase 5
DN99552_c7_g2_i2	2.32	Up	GDSL esterase/lipase
DN99552_c7_g4_i1	1.54	Up	GDSL esterase/lipase 2
DN99822_c2_g1_i3	-1.40	Down	GDSL esterase/lipase 1
**protein DETOXIFICATION**			
DN19383_c0_g1_i2	-3.22	Down	protein DETOXIFICATION
DN92995_c2_g1_i12	1.31	Up	protein DETOXIFICATION
DN94083_c5_g1_i6	3.43	Up	protein DETOXIFICATION 28
DN94507_c5_g1_i14	1.48	Up	protein DETOXIFICATION
DN94515_c7_g1_i1	-1.36	Down	protein DETOXIFICATION
DN95546_c0_g10_i2	-5.04	Down	protein DETOXIFICATION 48
DN95546_c0_g2_i1	1.44	Up	protein DETOXIFICATION
DN97141_c3_g1_i1	2.99	Up	protein DETOXIFICATION
DN97141_c3_g2_i2	3.62	Up	protein DETOXIFICATION 26
DN97550_c6_g1_i1	4.00	Up	protein DETOXIFICATION 40
DN98233_c1_g3_i6	-1.89	Down	protein DETOXIFICATION
DN98432_c5_g1_i2	-1.11	Down	protein DETOXIFICATION
DN98516_c6_g1_i1	4.25	Up	protein DETOXIFICATION 27
DN98841_c3_g1_i10	1.59	Up	protein DETOXIFICATION
**disease resistance protein**			
DN86530_c0_g1_i1	1.41	Up	disease resistance protein
DN94133_c4_g1_i2	1.50	Up	disease resistance protein RGA4
DN95060_c1_g1_i1	1.60	Up	disease resistance protein RGA2
DN95568_c3_g1_i1	1.32	Up	disease resistance protein RGA4
DN95848_c4_g4_i1	1.55	Up	disease resistance protein SUMM2
DN96494_c1_g1_i3	2.18	Up	disease resistance protein
DN96730_c4_g1_i1	-2.29	Down	disease resistance protein

DN98819_c5_g1_i3	-3.53	Down	disease resistance protein
DN99041_c5_g1_i1	-1.08	Down	disease resistance protein
DN99419_c3_g2_i1	2.35	Up	disease resistance protein RGA2
DN99775_c3_g6_i2	1.81	Up	disease resistance RPP13−like protein 1
**MLP−like protein**			
DN98392_c9_g2_i1	-1.00	Down	MLP−like protein 31
**protein IQ−DOMAIN**			
DN95107_c9_g1_i1	-1.71	Down	protein IQ−DOMAIN 1
DN98389_c5_g1_i1	-2.36	Down	protein IQ−DOMAIN 14
DN98749_c4_g1_i4	-1.51	Down	Protein IQ−DOMAIN 1
**Germin−like protein subfamily**			
DN93076_c5_g3_i1	-1.65	Down	germin−like protein subfamily 5 member 1
DN94529_c3_g1_i1	-1.90	Down	germin−like protein subfamily 3 member 1
DN95973_c5_g2_i1	-1.06	Down	germin−like protein subfamily 1 member 1
DN98779_c1_g2_i2	5.34	Up	germin−like protein subfamily 2 member 1

DEGs were differentially expressed with statistical significance ($|Log_2FC(Cd-T/CK)| \geq 1$ and *P*−value ≤ 0.05).
